# Supplementary material for: What matters to medical ward patients, and do we measure it? A qualitative comparison of patient priorities and current practice in quality measurement, on UK NHS medical wards
Source: BMJ Open. 2019 Mar 30;9(3):e024058. doi: 10.1136/bmjopen-2018-024058 (PMC6475203; doi:10.1136/bmjopen-2018-024058)
Supplement: Supplementary data [file bmjopen-2018-024058supp001.pdf]

TOPIC GUIDE: Current patient / carer for current patient

1. Let's find out about you.

Age
Employment
Education – school / university / postgraduate
Social support structures & marital status
Ethnicity

2. Why are you in hospital now?

Current diagnosis
Other conditions
Approximate length of stay to date
< 1 day
1 – 5 days
5 – 10 days
> 10 days

3. How many times have you been admitted to hospital in the last 6 months?

1-5
5-10
>10

4. Do you always come to this hospital or have you been admitted to other local hospitals?

5. How do you know if you're on a good ward? What is a 'good ward' to you?

Environment
- Clean
- Quiet
- Toilet and shower are available when required
- Meal timeliness, warmth
- Help available when requested
- Staff are responsiveness to my needs / my family's needs

Welcome
- My arrival is expected
- Staff introduce themselves
- Staff make me feel I will be well looked after; show a caring attitude; and don't rush me

Communication and use of personal information
- Accurate knowledge of previous medical history / current diagnosis / current investigations / discharge plan / medication reconciliation
- Quality of communication / teamwork

Discharge preparation

Friends' / families' recommendations

Ward information boards / quality and safety boards

Ward information leaflets / other printed materials.

Ward information displays / electronic screens

6. **If you had to decide whether a ward was good or not, what information would you need to make that decision?**

7. **Have you noticed any of the information the ward displays about itself? What do you think of the information you've seen?**

Friends and family test results
Safety cross
Shift-by-shift staffing
Falls
Pressure ulcers
Safety thermometer / harm-free care
Venous thromboembolism prophylaxis
Hand hygiene compliance
Hospital-acquired infections
Infection rates
Incident reporting

8. **What would you like to know about how your ward is performing?**

Hand hygiene compliance
Staffing levels
Friends and family results
Hospital-acquired infections
Pressure Ulcers
Falls
Venous thromboembolism prophylaxis
Complaints
Compliments
Length of stay
Mortality
Readmission rate
Safety climate

9. **How should your ward make that information available to you and your family??**

Ward displays
Leaflets
Smartphone / other device
Webpage

10. Preference for information seeking

Information-seeking sub-scale

	Disagree strongly	Disagree slightly	Neutral	Agree slightly	Agree strongly
As you become sicker you should be told more and more about your illness					
You should understand completely what is happening inside your body as a result of your illness					
Even if the news is bad, you should be well informed					
Your doctor should explain the purpose of your laboratory tests					
It is important for you to know all the side effects of your medication					
Information about your illness is as important to you as treatment					
When there is more than one method to treat a problem, you should be told about each one					

11. Have you previously had to complain about care or healthcare staff, nurses or doctors? What made you complain? How? PALS / informally / in writing?

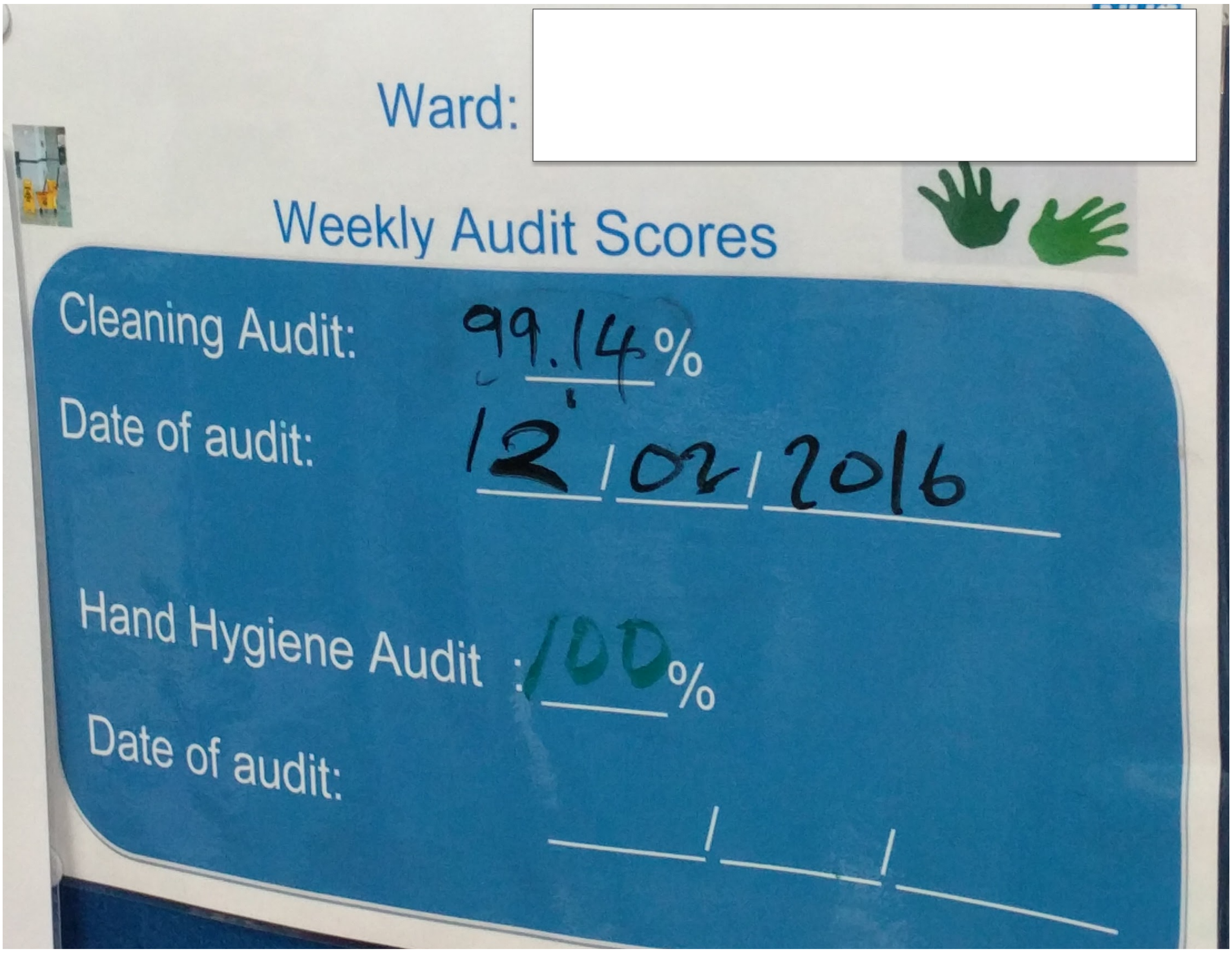
Ward:
Weekly Audit Scores
Cleaning Audit: 99.14%
Date of audit: 12/02/2016
Hand Hygiene Audit :100%
Date of audit: / /

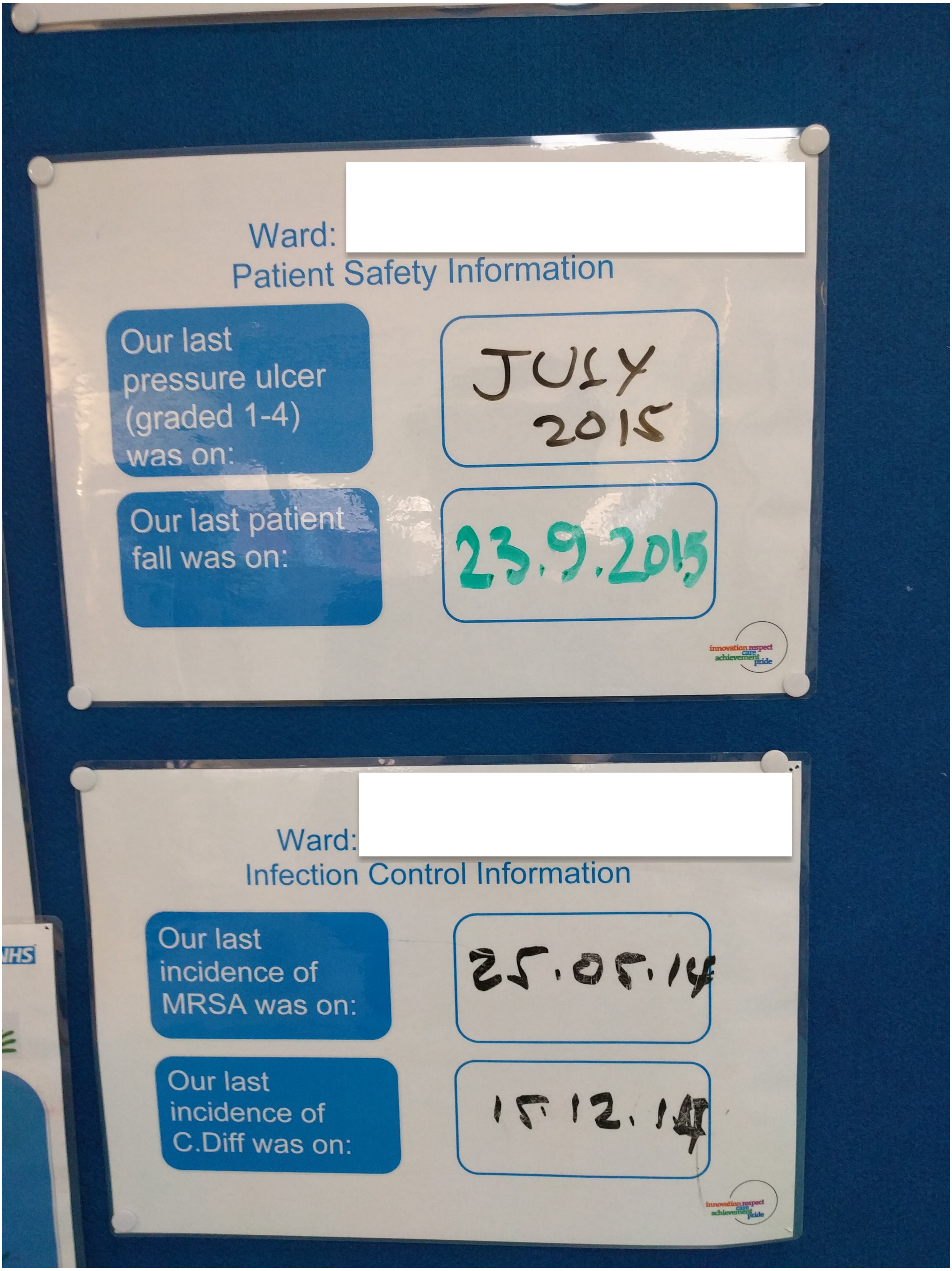
Ward:
Patient Safety Information
Our last pressure ulcer (graded 1-4) was on:
JULY 2015
Our last patient fall was on:
23.9.2015
Ward:
Infection Control Information
Our last incidence of MRSA was on:
25.05.14
Our last incidence of C.Diff was on:
15.12.14
NHS

[redacted]

I-Staff Safe Staffing Information Board

The Nurse in Charge today is: [redacted]

Staffing Status [redacted]

DATE: 15-2-15

Current Shift

	Staff required	On duty
Registered Nurses	1<br>3	1<br>1
Healthcare Assistants	2	2
Additional Support (for e.g. specials)		

Roles and Responsibilities

	Responsibilities
**Nurse in Charge**	Provides supervision, management and direct clinical care
**Registered Nurses**	Provides direct clinical care and supervision
**Health Care Assistants**	Provides direct care under the supervision of a registered nurse
**Additional Support**	Specials (may be registered or unregistered providing 121 direct care with supervision)

If you have any queries about the staffing on this ward please speak to the Nurse in Charge
