# Supplementary material for: What matters to medical ward patients, and do we measure it? A qualitative comparison of patient priorities and current practice in quality measurement, on UK NHS medical wards
Source: BMJ Open. 2019 Mar 30;9(3):e024058. doi: 10.1136/bmjopen-2018-024058 (PMC6475203; doi:10.1136/bmjopen-2018-024058)
Supplement: Supplementary file 2 [file bmjopen-2018-024058.draft_revisions.pdf]

BMJ Open is committed to open peer review. As part of this commitment we make the peer review history of every article we publish publicly available.

When an article is published we post the peer reviewers' comments and the authors' responses online. We also post the versions of the paper that were used during peer review. These are the versions that the peer review comments apply to.

The versions of the paper that follow are the versions that were submitted during the peer review process. They are not the versions of record or the final published versions. They should not be cited or distributed as the published version of this manuscript.

BMJ Open is an open access journal and the full, final, typeset and author-corrected version of record of the manuscript is available on our site with no access controls, subscription charges or pay-per-view fees (http://bmjopen.bmj.com).

If you have any questions on BMJ Open's open peer review process please email info.bmjopen@bmj.com

BMJ Open

What do medical ward patients want, and do they get it? A comparison of patient priorities in care quality and current practice in quality measurement

Journal:	*BMJ Open*
Manuscript ID	bmjopen-2018-024058
Article Type:	Research
Date Submitted by the Author:	08-May-2018
Complete List of Authors:	Pannick, Samuel; Charing Cross Hospital, Gastroenterology<br>Archer, Stephanie; Imperial College London, NIHR Imperial Patient Safety Translational Research Centre; Imperial College London,<br>Long, Susannah; Imperial College London, Centre for Patient Safety and Service Quality<br>Husson, Fran; Imperial College, Faculty of Medicine<br>Athanasiou, Thanos; Imperial College London, Surgery and Cancer<br>Sevdalis, Nick; King's College London,
Keywords:	Quality in health care < HEALTH SERVICES ADMINISTRATION & MANAGEMENT, GENERAL MEDICINE (see Internal Medicine), Organisation of health services < HEALTH SERVICES ADMINISTRATION & MANAGEMENT, Clinical governance < HEALTH SERVICES ADMINISTRATION & MANAGEMENT

SCHOLARONE™
Manuscripts

only

What do medical ward patients want, and do they get it?

A comparison of patient priorities in care quality and current practice in quality measurement

Samuel Pannick,[1,2] Stephanie Archer,[1] Susannah J Long,[1,2] Fran Husson,[1,2]

Thanos Athanasiou,[3] & Nick Sevdalis[4]

[1] NIHR Imperial Patient Safety Translational Research Centre, Imperial College London, UK

[2] Imperial College Healthcare NHS Trust, London, UK

[3] Department of Surgery & Cancer, Imperial College London, UK

[4] Centre for Implementation Science, King's College London, UK

Corresponding author:

Dr Sam Pannick, MA MB BS MRCP PhD

Specialist Registrar in Gastroenterology and Internal Medicine, Department of Gastroenterology, Charing Cross Hospital, Fulham Palace Road, W6 8RF

United Kingdom

sam.pannick@nhs.net; + (44) 203 311 1234

For peer review only

Author information:

Dr Stephanie Archer, Research Fellow, NIHR Imperial Patient Safety Translational Research Centre, Imperial College London.

Dr Susannah J Long, Consultant Geriatrician, Imperial College Healthcare NHS Trust, and Honorary Clinical Senior Lecturer, NIHR Imperial Patient Safety Translational Research Centre, Imperial College London.

Ms Fran Husson, Patient Advocate, Imperial College Healthcare NHS Trust, and Honorary Research Officer, Imperial College London.

Professor Thanos Athanasiou, Consultant Cardiothoracic Surgeon, Imperial College Healthcare NHS Trust, and Professor of Cardiovascular Sciences and Cardiac Surgery, Imperial College London.

Professor Nick Sevdalis, Professor of Implementation Science and Patient Safety, King's College London.

Word count: 3133

Conflicts of interest: This paper represents independent research supported by the National Institute for Health Research (NIHR) Imperial Patient Safety Translational Research Centre, and the Imperial College Healthcare Charity (Grant GG14\1022). NS' research is supported by the NIHR Collaboration for Leadership in Applied Health Research and Care South London at King's College Hospital NHS Foundation Trust. NS is a member of King's Improvement Science, which is part of the NIHR CLAHRC South London and comprises a specialist team of improvement scientists and senior researchers based at King's College London. Its work is funded by King's Health Partners (Guy's and St Thomas' NHS Foundation Trust, King's College Hospital NHS Foundation Trust, King's College London and South London and

For peer review only - http://bmjopen.bmj.com/site/about/guidelines.xhtml

Maudsley NHS Foundation Trust), Guy's and St Thomas' Charity, the Maudsley Charity and the Health Foundation. No funding source had any role in the design and conduct of the study; collection, management, analysis or interpretation of the data; or preparation, review or approval of the manuscript. The views expressed are those of the authors and not necessarily those of the NHS, the NIHR or the Department of Health and Social Care.

Contributions:

Study design: SP, SA, TA, FH, NS, SJL. Study implementation and data collection: SA, SP, SJL. Analysis: SA, SP, NS, FH, TA. All authors contributed to, read and approved the final manuscript.

Ethical approval:

Ethical approval was granted by the Westminster Research Ethics Committee (16/LO/0196) and the hospital's joint research compliance office (16SM3129).

Acknowledgements: We are grateful to Drs Louise Hull and Tayana Soukup for their help in facilitating the focus groups. We thank Ms Margaret Turley (carer representative) from the NIHR Imperial PSTRC for her help developing the interview guide.

Data sharing: There are no additional unpublished data.

Key words: healthcare quality; medical ward; patient experience

For peer review only

ABSTRACT

Objectives

To compare the quality metrics selected for public display in NHS medical wards to patients' and carers' expressed quality priorities.

Design

Observational assessment of general medical ward practice and semi-structured interviews.

Setting

UK tertiary hospital

Participants

Fourteen patients and carers on acute medical wards and geriatric wards.

Results

Quality metrics on public display evaluated hand hygiene, hospital-acquired infections, nurse staffing, pressure ulcers, falls, and patient feedback. The intended audience for these metrics was unclear, and the displays gave no indication as to whether performance was improving or worsening. Interviews identified three perceived key components of high quality ward care: communication, staff attitudes, and hygiene. These aligned poorly with the priorities on display. Incomplete performance reporting had the potential to reduce patients' trust in their medical teams. More philosophically, patients' and carers' ongoing experiences of care would override any other evaluation, and they felt little need for measures relating to previous performance. The display of performance reports only served to emphasise patients' and carers' lack of control in this inpatient setting.

For peer review only

Conclusions

There is a persistent gap between general medical inpatients' care priorities and the aspects of care that are publicly reported. Patients and carers do not act as 'informed choosers' of healthcare in the inpatient setting, and tokenistic quality measurement may have unintended consequences.

STRENGTHS AND LIMITATIONS OF THE STUDY

- The study highlights the differences between inpatients' views of care quality and the care priorities expressed through public performance reporting.
- Participants included older, frail patients, and those who did not speak English as a first language – demographics often excluded from safety and quality research.
- The 'static' performance measures seen at the study site are typical of those reported in other literature.
- Other repositories for quality metrics, beyond the ward displays analysed here, may better approximate patient priorities.

INTRODUCTION

Patient involvement is a priority for the patient safety and healthcare quality movement,[1] but how best to involve patients remains unclear. Policymakers favour the transparent publication of quality metrics as a means of engaging patients in their care, framing this engagement as an informed choice of healthcare provider. In the right context, providing appropriate information can improve patients' and carers' participation in their care, perhaps even improving outcomes.[2] In the UK, this 'informed choice' argument has led to the mandatory display of performance metrics on NHS inpatient wards.[3]

For peer review only - http://bmjopen.bmj.com/site/about/guidelines.xhtml

In the acute setting, however, inpatients are unlikely to use performance measures as would typical 'consumers'. Few choices are available. The debilitation and stress of an acute illness can impede information processing, and intense anxiety can lead to active information avoidance. This may equally affect patients' families or carers, who focus on the immediate health concerns of their loved ones. Even when patients are comfortable accessing complex information at home, they should be treated as 'situationally-impaired' in the hospital environment.[4] Whether inpatients value service-level metrics, and how they relate to them, have not yet been evaluated.

Here, we compare the quality metrics selected for public display in NHS medical wards to patients' and carers' expressed quality priorities. We aim to capture patients' and carers' perceptions of a 'good ward', and evaluate their reactions to the quality metrics on display. Our secondary aim was to identify a set of quality metrics which might align incentives for the varied stakeholders on these units – including staff, managers and patients.

METHODS

The study was conducted on general medical wards, which provide the majority of acute inpatient care but struggle for organisational attention or targeted improvement strategies.[5] We assessed ward information displays in acute medical wards and geriatric wards at a tertiary hospital in London, with a standardised instrument. Free text notes highlighted any adjacent information on the display boards. Examples were photographed with a digital camera. Photos were then used as prompts in semi-structured interviews with general medical inpatients and their carers at the hospital. The interviews were based on a topic guide, co-developed with patient and carer representatives, exploring care priorities and the concept of a 'good ward' [**Online supplement**].

For peer review only

Ward staff (doctors, nurses, and allied professionals) were asked to suggest patients or carers who would be physically capable of taking part in an interview. Participants were aged over 18 years, and able to provide informed consent. Patients were excluded if they were physiologically unstable, had major cognitive or communication difficulties, or did not speak English. The interviews took place at patients' bedsides, as described previously in qualitative work with hospitalised medical patients.[6] The interviews were conducted by a specialist registrar in internal medicine and gastroenterology, undertaking a PhD in healthcare quality improvement, with previous experience of qualitative research (SP). Interviews were audiotaped, and then transcribed verbatim. Using NVivo (QSR International, Australia), two researchers (SP and SA) analysed the transcripts using an inductive thematic analysis.[7] Data collection ceased when the study reached saturation, with no new themes emerging. Other qualitative interview studies reached data saturation within the first 12 interviews.[8]

Ethical approval was granted by the Westminster Research Ethics Committee (16/LO/0196) and the hospital's joint research compliance office (16SM3129).

Patient involvement

The interview topic guide was co-produced with local patient and carer representatives, who in turn canvassed their patient and carer networks for opinions and feedback. The patient representative (FH) co-authored the final manuscript reporting the study's results.

RESULTS

Interview participants

Fourteen people were interviewed (nine patients and five carers). Seven were female. Patients had a median age of 75 (range 57-86), with a median length of stay of five days.

For peer review only - http://bmjopen.bmj.com/site/about/guidelines.xhtml

71% of participants spoke English as a first language. Half of the patients depended on family or community support, and one-third of them had undergone other hospital admissions in the preceding six months. Nine interviews took place on the acute medical wards and five on medicine for the elderly wards. Interviews lasted a median of 23 minutes (range 11-48 minutes).

What performance metrics were on display, and how were they portrayed?

Performance metrics evaluated hand hygiene, hospital-acquired infections (MRSA and C.difficile), nurse staffing, pressure ulcers, falls, and patient feedback [**Online supplement & Table 1**]. The intended audience for these metrics was often unclear: individual display boards contained combinations of messages for patients and staff. Possessive pronouns (our and your) and pronouns (we and you) were used interchangeably, within the same display, to refer to both patients and staff.

Performance measures were displayed with little background information or context. Each metric was displayed as a single, static measure of performance, with no evidence of trends over time. There was no indication of an acceptable benchmark. No patient-actionable information was given for any of the performance measures, other than a suggestion to speak to a senior nurse for more information about staffing on the ward. Ward displays about local quality and safety priorities (e.g., 'MRSA compliance') were not explicitly linked to previous performance.

Thematic analysis: what makes a 'good ward' in the eyes of the patients and their carers?

The interviews identified three key components of high quality ward care: communication, staff attitudes, and hygiene.

For peer review only

1. Communication

Participants felt entirely dependent on staff to keep them abreast of forthcoming investigations and treatments. They valued prompt communication and were keenly aware of its absence. At the same time, they recognised that treatment plans would frequently change, often for reasons outside of their teams' control, and simply held those teams accountable for keeping them updated:

> *'I know it is not always possible that definitive information is available. But as long as you are informed to the ability that they can inform you, you cannot have any gripes about that. If someone says to you, "Look, you may go home tomorrow", I am big enough and ugly enough to know that it may be the day afterwards…' (Patient 3)*

The value of effective, shared communication within the multidisciplinary team was also highlighted. The capacity to speak to one team member, and have that conversation disseminated promptly to the rest of the team, was a key feature of good performance:

> *'I have found you'll be speaking to one person – and it could be a nurse or a doctor or anybody else – and at the end of the day, everybody knows what I'm talking about... So you can communicate with [just] one person... It's a vital thing.' (Carer 1)*

Most comments about information sharing within multidisciplinary teams came from carers, rather than patients. This perhaps reflected the role of carers in the ward environment, where they act both as an information source for professional teams and as advocates for the patients.

2. Staff attitudes

For peer review only

The second element of high quality care was staff attitude. Considerable attention was paid to *how* staff went about their work: staff attentiveness, or 'service', influenced whether patients felt they were on a good ward. Adjectives like 'jolly', 'respectful' and 'helpful', or 'abrupt' and 'wishy-washy', were not so much seen as individual personality attributes, as they were features of work performance:

> *'[A] good ward is to be helpful to patients, being more human than a machine, you understand?' (Patient 2)*

> *'I think it's the attitude of people [that makes a good ward]. It's the main thing.' (Patient 6)*

Thus, the manner of care delivery – rather than the resources available for it – largely defined the care experience. The corollary of this was the potential for a major change between one shift and the next, even on the same ward. There was a sense, perhaps, that rather than a good or bad ward there were just good shifts or bad shifts:

> *'Where it changes more than anything else is at night, when you have a complete change of staff. Sometimes the night staff that come on are absolutely fantastic, and are very engaged. But sometimes they are entirely the opposite. It is like, "Well we are just here to get you through until the morning, when the people that are looking after you come back."' (Patient 3)*

As well as analysing their own interactions with staff, patients and carers were keen observers of the working relationships between different professionals on the ward.

For peer review only - http://bmjopen.bmj.com/site/about/guidelines.xhtml

Whether staff seemed appreciative of each other's efforts, or were openly disrespectful to one another, caused patients to wonder how they too were being treated:

> *'[You might think] the more staff, the better the person feels, and that is not how I feel... Everything depends on the lower level[s] of staff we've got working in the ward... and their position [should be] respected by the doctors and the more senior people... They did all [sorts of tasks], and nobody seemed to recognise that they were doing something like that...' (Patient 7)*

> *'Two nurses were having a fight with each other, and that's not very good for the rest of us. And of the course the supervisor was asking them to be quiet, because they were shouting and screaming at each other.' (Patient 4)*

Yet these observations of staff behaviours were quite nuanced. Patients recognised different types of unproductive working relationships, describing over-familiarity (*'almost like a bunch of friends working together' – Patient 3*), as well as open antagonism. They also made allowances for the general workload on the ward, even excusing displays of inappropriate behaviour:

> *'There's a lot of pressure put on the staff, you know it's understandable. You can see that they're actually very tired people, they needed a good rest, and that's why the whole thing gets on top of them, they're overworked.' (Patient 4)*

3. <u>Hygiene</u>

In a similar vein, patients and carers expressed quite subtle views of why they held hygiene standards to be important. First, good hygiene was *de facto* evidence of a ward that was

For peer review only

providing safe care, with little risk of iatrogenic infection. Patients and carers were conscious of the possibility of hospital-acquired infection, understanding it as a major risk associated with inpatient care. Minimising that risk made it possible to focus on the acute medical issues at hand. Second, good hygiene served as a deeper marker of staff pride, diligence and attention to detail, all of which were reassuring:

> *'The cleanliness aspect, I think, is... more important than possibly people realise... It sets out a marker if you like... if the mindset of the ward is, you know, "We are proud of the place that we work in." So it is a fairly good marker of how that ward will actually be.' (Patient 3)*

Thematic Analysis: How did patients and carers perceive the quality metrics on display?

1. <u>Benefits – using infection data for hand hygiene, and understanding staff performance</u>

Patients and carers described some benefits of the quality metrics on display, particularly when it came to infection data. They acknowledged prompts to focus on their own hand hygiene, whilst hoping that staff would do the same. In some cases, a vague familiarity with infection control terminology was helpful:

> *'Because that's in the press [MRSA rates], I suppose people do want to know that, don't they? All of this you read in the papers of people being in hospital - they went in with one thing and they came out with that... You don't want to get worse. They're meant to be making you better.' (Patient 5)*

Real-time information on staffing levels was also potentially helpful, in that it could help set realistic expectations of the care patients might receive:

For peer review only - http://bmjopen.bmj.com/site/about/guidelines.xhtml

'When I'm getting poor service on a particular day, at least I can see that there might be a good reason for it... I would be more understanding, if I had to wait twice as long for help, if I knew that there was only half the number of staff there should be.' (Patient 8)

'When I saw the amount of staff that you're supposed to have on the ward, there were not half the staff. So the other staff that turned up were constantly busy, running back and forth, and you can see how much stress they were [under]. But they were doing a good job... You can see the nurse who has turned up is doing a really good job.' (Carer 1)

2. Significant drawbacks – problems in information delivery, prioritisation of personal experience, and unintended consequences

However, patients and carers were largely disparaging about the quality metrics on display. There were numerous problems with information delivery, such as inadequate font size or colour contrast. Yet even with these issues addressed, the information provided was fundamentally inadequate to make a judgement about quality. Patients struggled to see the relevance of a single figure when no trend or benchmark was provided:

'Obviously as a member of the public I want the minimum [information], but I have nothing to compare it with. So if you [say], "We've not had one [infection] for three years", I can't compare that with anything. So it doesn't mean anything to me...' (Carer 5)

For peer review only - http://bmjopen.bmj.com/site/about/guidelines.xhtml

'That [single figure] doesn't mean anything. That doesn't inform. It could be an increase... but it could be [a] decrease.' (Patient 2)

More broadly, participants felt little need for measures that related to their wards' previous performance. Their ongoing experiences of care would override any other evaluation. From each individual's perspective, their personal care was the priority, whether or not it reflected a typical standard of care on that ward. In that light, other performance metrics became irrelevant:

'I use my own judgement. If I'm satisfied: that's it.' (Patient 9)

'If we want information, we ask for it and we get it. As long as [my relative] is alright and getting looked after, I'm not really bothered about nothing else. If she's getting well looked after, the nurses are lovely, their care is great... that's all we are concerned about.' (Carer 3)

As a result, the production of ward quality metrics had some unintended consequences, even going so far as to reduce patients' trust in the whole enterprise. The absence of baseline data in quality displays in particular raised suspicions that poor performance was being concealed.

'Let us face it... you have got your 100% figure there. Would you put up a 20% figure? ... What would you be doing? You would be ruining the confidence of the patients...' (Patient 3)

For peer review only - http://bmjopen.bmj.com/site/about/guidelines.xhtml

Patients and carers felt that staff had to have ownership of the quality agenda in hospital: quality metrics were for staff – not patients – to digest. Many interviewees drew comparisons with other settings in which they were consumers: as restaurant diners, or as car purchasers, where their ability to exercise a choice was crucial. Here, however, they had no power to choose, and the display of performance reports only served to emphasise their lack of control:

> *'It would be great if I'm admitted and I'm given a choice of five wards, and I would say, "Well, how do I know which one's which, which one's best?" My next question would be, "Can you give me the audits of those wards to show which has the highest rating?" and I would go to that... If there's no choice, then it's all academic.' (Patient 8)*

> *'Well, other than clean the wards, there's not a lot we can do is there? What else can you do?' (Carer 4)*

The 'Friends and Family Test' question was found to be particularly challenging, given that these patients had no choice in arriving on the ward in the first place, nor could subsequent patients exercise a preference to get there. Indeed, the service pressures on hospital admissions were so well publicised that the idea of choosing a ward seemed faintly ridiculous:

> *'Would I recommend a ward? How can you recommend a ward?... I mean, that's a daft question, because... they put you in the place you need to be, don't they?' (Patient 5)*

For peer review only

DISCUSSION

To our knowledge, this is the first study to compare publicly-displayed performance metrics with patient and carer perceptions of high quality care on UK medical wards. We identified discrepancies between patient- and carer-identified priorities and the quality metrics relating to their care on general medical wards. Three core components of high quality general medical care (communication, staff attitudes, and hygiene) were only partially aligned with the performance measures on display. Specifically, we found process and outcome measures relating to hand hygiene and iatrogenic infection, but none specifically relating to attitudes or communication. Patients and carers acknowledged limited benefits to the display of performance data, but had significant reservations about how it was contextualised. They relied on their own experience of care to judge its quality, above any objective measure of performance. More philosophically, they questioned the purpose of publicly displayed performance data, given their lack of choice in this setting. In some cases, these reservations actually eroded trust in ward teams' performance.

This study builds on a body of research exploring patient priorities and patient involvement in the acute hospital setting. Boyd surveyed recently-discharged patients, similarly finding that communication, patient-professional interactions, hygiene and the technical delivery of care were their main priorities.[10] Our study suggests that Boyd's findings (which excluded current inpatients) were not unduly affected by recall bias. Nonetheless, hospitalised patients remain relatively indifferent to service-level performance and change.[11] We suggest an explanation for this: current inpatients are unable to exercise informed choices about their ward, nor are they able to directly use information to improve performance. They are therefore excluded from the two key pathways by which performance measurement may lead to quality improvement.[12]

For peer review only - http://bmjopen.bmj.com/site/about/guidelines.xhtml

Our findings support the recent call for the abolition of the mandatory 'Friends and family test'.[13] It is expensive to maintain, "at best tolerated, often ignored, and sometimes ridiculed".[13] The resulting tokenistic display of performance data erodes patients' trust in their caregivers. It can also be corrosive for staff morale, both at the frontline and at board level.[14,15] This tokenism is perpetuated by a dearth of resources for implementing improvement.[1,16] A credible, co-produced, quality framework for acute medical inpatients is urgently required, with outcomes that are sensitive to the work[17] and structures[18] of inpatient care. Co-produced quality standards should capitalise on the active contributions of patients and carers, rather than depicting them as 'informed choosers' of healthcare provision.

Study limitations include a relatively small sample from a single site. Nonetheless, the group of interviewees was a representative one, and the study reached data saturation. Participants included older, frail patients, and a significant proportion did not speak English as a first language – demographics often excluded from safety and quality research.[19] Ward displays were also representative: the use of 'static' performance measures, as seen here, is widespread.[20] There are other repositories for quality metrics, beyond those ward displays analysed here, which may better approximate patient priorities. However, they typically use composites of the data we found,[21] or are aggregated to the hospital level, with no ward-level interpretation.[22,23]

CONCLUSION

There is a persistent gap between general medical inpatients' care priorities and the aspects of care that are publicly reported. Tokenistic quality measurement may have unintended consequences, eroding patients' trust in ward teams.

For peer review only

REFERENCES

1. Lawton R, O'Hara JK, Sheard L, et al. Can patient involvement improve patient safety? A cluster randomised control trial of the Patient Reporting and Action for a Safe Environment (PRASE) intervention. BMJ Qual Saf 2017;**26**(8):622-31.
2. Shaller D, Kanouse DE, Schlesinger M. Context-based strategies for engaging consumers with public reports about health care providers. Med Care Res Rev 2014;**71**(5 Suppl):17S-37S.
3. Department of Health. Hospital 'friends and family test' announced. 2012. https://www.gov.uk/government/news/hospital-friends-and-family-test-announced. Accessed 11/12/2017.
4. Morris D, Karlson A. Dynamic Accessibility Requirements for Hospital Patients. SIGCHI Conference on Human Factors in Computing Systems. Vancouver, Canada: ACM Press, 2011.
5. Pannick S, Wachter RM, Vincent C, et al. Rethinking medical ward quality. BMJ 2016;**355**:i5417.
6. Tobiano G, Bucknall T, Marshall A, et al. Patients' perceptions of participation in nursing care on medical wards. Scand J Caring Sci 2016;**30**(2):260-70.
7. Braun V, Clarke V. Using thematic analysis in psychology. Qualitative Research in Psychology 2006;**3**(2):77-101.
8. Guest G, Bunce A, Johnson L. How Many Interviews Are Enough?: An Experiment with Data Saturation and Variability. Field Methods 2006;**18**(1):59-82.
9. NHS England. Friends and Family Test: Guidance. 2014. Publications Gateway Ref No. 01787.
10. Boyd J. The 2006 inpatients importantance study. Oxford: Picker Institute, 2007. http://www.nhssurveys.org/Filestore/documents/Findings_and_development_of_the_2006_Inpatients_Importance_study_final.pdf.
11. Wray CM, Farnan JM, Arora VM, et al. A qualitative analysis of patients' experience with hospitalist service handovers. J Hosp Med 2016;10.1002/jhm.2608.
12. Berwick DM, James B, Coye MJ. Connections between quality measurement and improvement. Med Care 2003;**41**(1 Suppl):I30-8.
13. Robert G, Cornwell J, Black N. Friends and family test should no longer be mandatory. BMJ 2018;**360**:k367.
14. NHS England. Review of the Friends and Family Test. 2014. https://www.england.nhs.uk/wp-content/uploads/2014/07/fft-rev1.pdf. Accessed 16/10/2017.
15. Raleigh V, Thompson J, Jabbal J, et al. Patients' experience of using hospital services. London: The King's Fund, 2015. http://www.pickereurope.org/wp-content/uploads/2015/12/Patients-experience-Kings-Fund-Dec-2015.pdf.
16. Sheard L, Marsh C, O'Hara J, et al. The Patient Feedback Response Framework - Understanding why UK hospital staff find it difficult to make improvements based on patient feedback: A qualitative study. Soc Sci Med 2017;**178**:19-27.
17. Pannick S, Davis R, Ashrafian H, et al. Effects of Interdisciplinary Team Care Interventions on General Medical Wards: A Systematic Review. JAMA Intern Med 2015;**175**(8):1288-98.
18. Dumitrascu AG, Burton MC, Dawson NL, et al. Patient portal use and hospital outcomes. Journal of the American Medical Informatics Association 2018;**25**(4):447-53.

For peer review only - http://bmjopen.bmj.com/site/about/guidelines.xhtml

19. O'Hara JK, Lawton RJ. At a crossroads? Key challenges and future opportunities for patient involvement in patient safety. BMJ Qual Saf 2016;10.1136/bmjqs-2016-005476.

20. Anhoj J, Hellesoe AB. The problem with red, amber, green: the need to avoid distraction by random variation in organisational performance measures. BMJ Qual Saf 2016;10.1136/bmjqs-2015-004951.

21. NHS Quality Observatory. NHS Safety Thermometer. https://www.safetythermometer.nhs.uk/index.php?option=com_content&view=article&id=1&Itemid=101. Accessed 24th June 2016.

22. Care Quality Commission. NHS Surveys: focused on patients' experience. 2016. http://www.nhssurveys.org/. Accessed 23rd June 2016.

23. Centers for Medicare and Medicaid Services. HCAHPS Fact Sheet. June 2015. Baltimore, MD. http://www.hcahpsonline.org/facts.aspx. Accessed 23rd June 2016.

For peer review only

For peer review only - http://bmjopen.bmj.com/site/about/guidelines.xhtml

Table 1: Performance indicators available in public ward areas

Performance indicator	*Display choice*	*Display format*
Hand hygiene	Percentage in last audit	Most recent result only; numerator and denominator definitions not provided.
Hospital-acquired infections	Date of last recorded event	Most recent result only
Pressure ulcers	Date of last recorded event	Most recent result only
Falls	Date of last recorded event	Most recent result only
Nurse staffing	Numbers of staff required for the shift *vs* those actually on duty, for staff nurses and health care assistants	Most recent result only; explanation of staff responsibilities
Patient feedback	'Friends and Family Test' star rating; percentage of patients who would recommend the ward*	Most recent result only; examples of patients' comments; no explanation of star rating system

*The 'Friends and Family Test' asks *"How likely are you to recommend our service to friends and family if they needed similar care or treatment?"*[9]

For peer review only

TOPIC GUIDE: Current patient / carer for current patient

1. Let's find out about you.

Age
Employment
Education – school / university / postgraduate
Social support structures & marital status
Ethnicity

2. Why are you in hospital now?

Current diagnosis
Other conditions
Approximate length of stay to date

< 1 day
1 – 5 days
5 – 10 days
> 10 days

3. How many times have you been admitted to hospital in the last 6 months?

1-5
5-10
>10

4. Do you always come to this hospital or have you been admitted to other local hospitals?

5. How do you know if you're on a good ward? What is a 'good ward' to you?

Environment

- Clean
- Quiet
- Toilet and shower are available when required
- Meal timeliness, warmth
- Help available when requested
- Staff are responsiveness to my needs / my family's needs

Welcome

- My arrival is expected
- Staff introduce themselves
- Staff make me feel I will be well looked after; show a caring attitude; and don't rush me

Communication and use of personal information

- Accurate knowledge of previous medical history / current diagnosis / current investigations / discharge plan / medication reconciliation
- Quality of communication / teamwork

Discharge preparation

For peer review only

Friends' / families' recommendations

Ward information boards / quality and safety boards

Ward information leaflets / other printed materials.

Ward information displays / electronic screens

6. If you had to decide whether a ward was good or not, what information would you need to make that decision?

7. Have you noticed any of the information the ward displays about itself? What do you think of the information you've seen?

Friends and family test results
Safety cross
Shift-by-shift staffing
Falls
Pressure ulcers
Safety thermometer / harm-free care
Venous thromboembolism prophylaxis
Hand hygiene compliance
Hospital-acquired infections
Infection rates
Incident reporting

8. What would you like to know about how your ward is performing?

Hand hygiene compliance
Staffing levels
Friends and family results
Hospital-acquired infections
Pressure Ulcers
Falls
Venous thromboembolism prophylaxis
Complaints
Compliments
Length of stay
Mortality
Readmission rate
Safety climate

9. How should your ward make that information available to you and your family??

Ward displays
Leaflets
Smartphone / other device
Webpage

For peer review only

10. Preference for information seeking

Information-seeking sub-scale

	Disagree strongly	Disagree slightly	Neutral	Agree slightly	Agree strongly
As you become sicker you should be told more and more about your illness					
You should understand completely what is happening inside your body as a result of your illness					
Even if the news is bad, you should be well informed					
Your doctor should explain the purpose of your laboratory tests					
It is important for you to know all the side effects of your medication					
Information about your illness is as important to you as treatment					
When there is more than one method to treat a problem, you should be told about each one					

11. Have you previously had to complain about care or healthcare staff, nurses or doctors? What made you complain? How? PALS / informally / in writing?

For peer review only

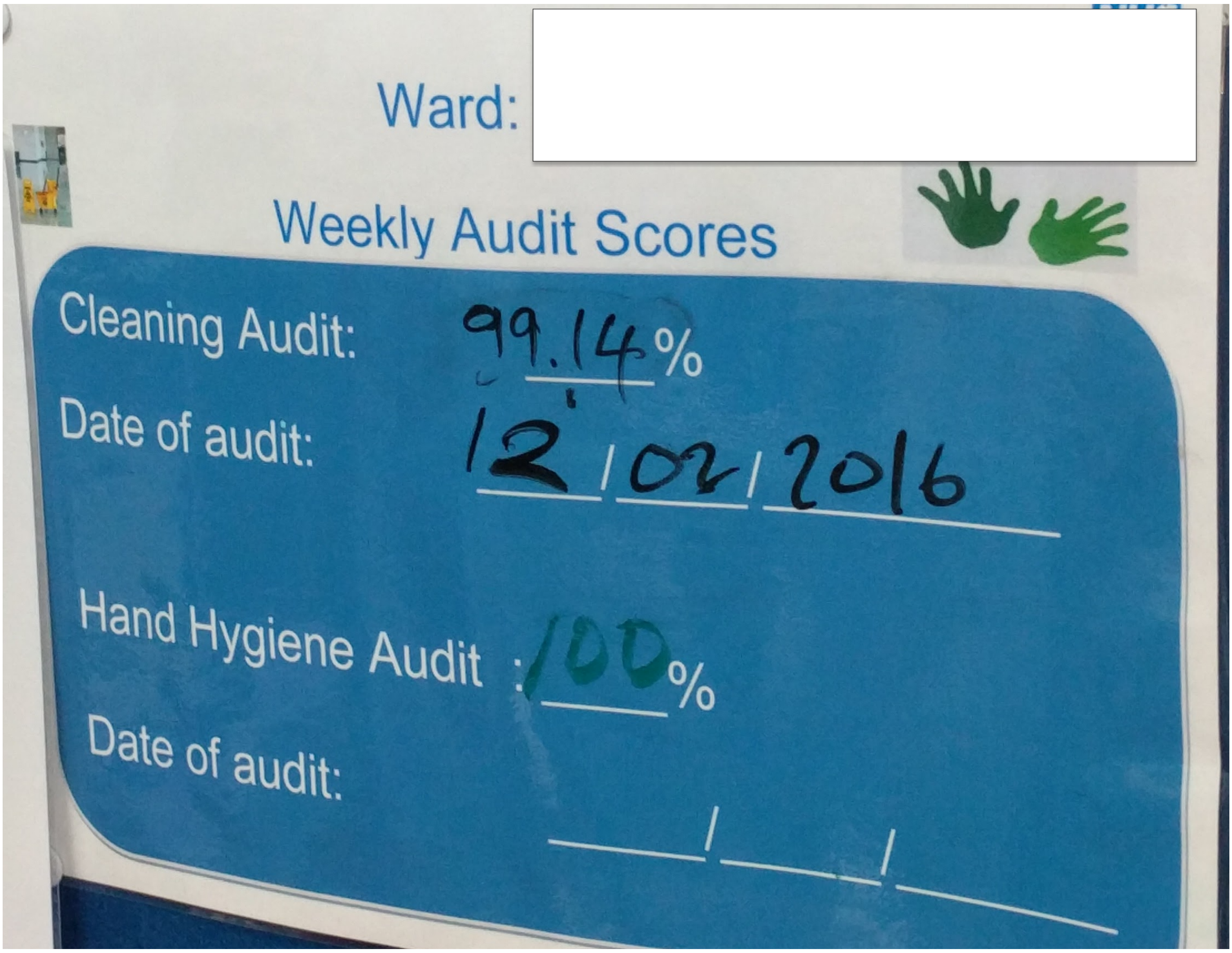
Ward:
Weekly Audit Scores
Cleaning Audit: 99.14%
Date of audit: 12/02/2016
Hand Hygiene Audit :100%
Date of audit:

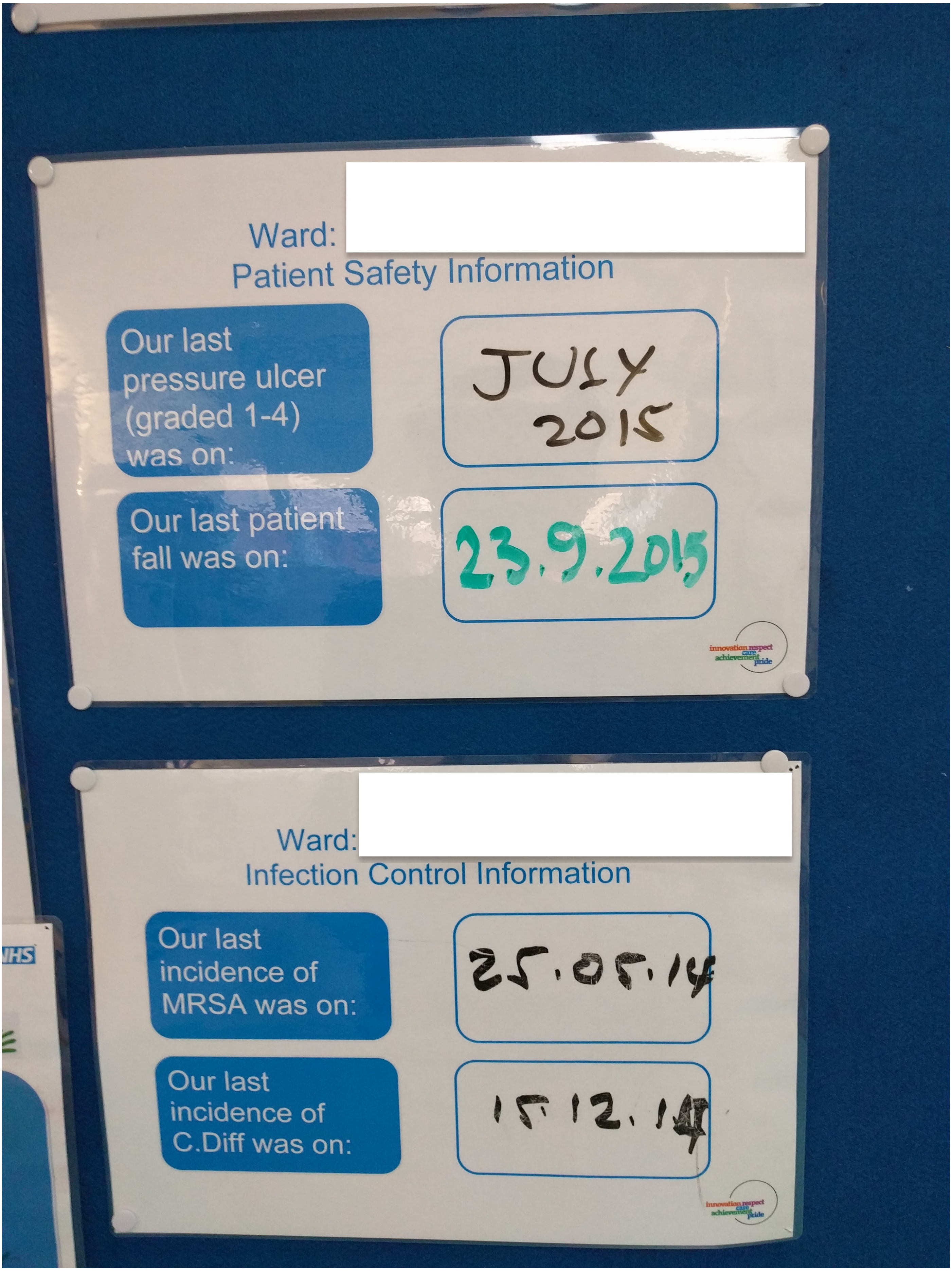
Ward:
Patient Safety Information
Our last pressure ulcer (graded 1-4) was on:
JULY 2015
Our last patient fall was on:
23.9.2015
Ward:
Infection Control Information
Our last incidence of MRSA was on:
25.05.14
Our last incidence of C.Diff was on:
15.12.14

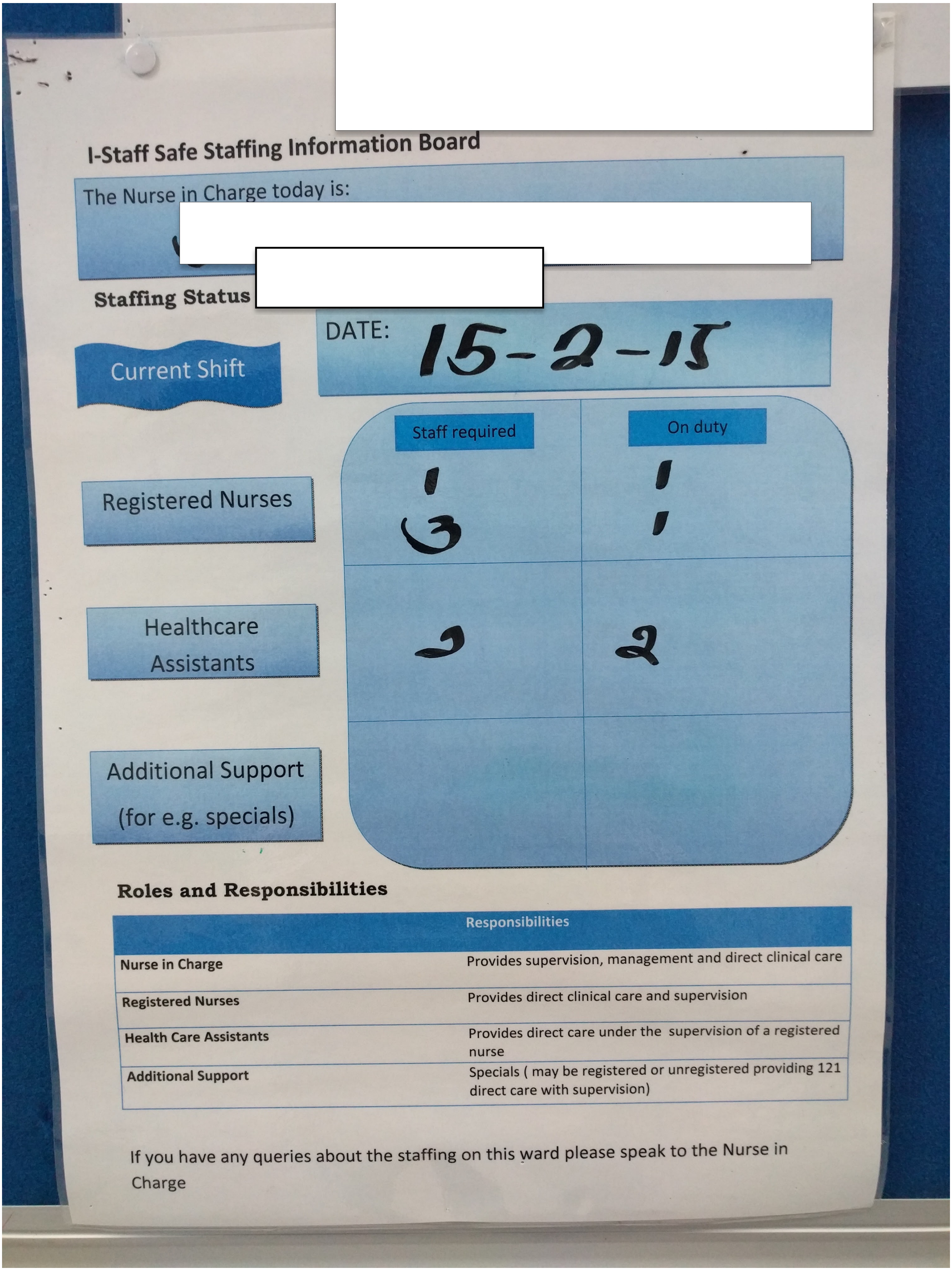
I-Staff Safe Staffing Information Board
The Nurse in Charge today is:
Staffing Status
Current Shift
DATE: 15-2-15
Staff required
On duty
Registered Nurses
1
3
1
1
Healthcare Assistants
2
2
Additional Support (for e.g. specials)
Roles and Responsibilities
Responsibilities
Nurse in Charge
Provides supervision, management and direct clinical care
Registered Nurses
Provides direct clinical care and supervision
Health Care Assistants
Provides direct care under the supervision of a registered nurse
Additional Support
Specials (may be registered or unregistered providing 121 direct care with supervision)
If you have any queries about the staffing on this ward please speak to the Nurse in Charge

COREQ (COnsolidated criteria for REporting Qualitative research) Checklist

A checklist of items that should be included in reports of qualitative research. You must report the page number in your manuscript where you consider each of the items listed in this checklist. If you have not included this information, either revise your manuscript accordingly before submitting or note N/A.

Topic	Item No.	Guide Questions/Description	Reported on Page No.
**Domain 1: Research team and reflexivity**			
Personal characteristics			
Interviewer/facilitator	1	Which author/s conducted the interview or focus group?	7
Credentials	2	What were the researcher's credentials? E.g. PhD, MD	7
Occupation	3	What was their occupation at the time of the study?	7
Gender	4	Was the researcher male or female?	7
Experience and training	5	What experience or training did the researcher have?	7
Relationship with participants			
Relationship established	6	Was a relationship established prior to study commencement?	7
Participant knowledge of the interviewer	7	What did the participants know about the researcher? e.g. personal goals, reasons for doing the research	7
Interviewer characteristics	8	What characteristics were reported about the inter viewer/facilitator? e.g. Bias, assumptions, reasons and interests in the research topic	7
**Domain 2: Study design**			
Theoretical framework			
Methodological orientation and Theory	9	What methodological orientation was stated to underpin the study? e.g. grounded theory, discourse analysis, ethnography, phenomenology, content analysis	7
Participant selection			
Sampling	10	How were participants selected? e.g. purposive, convenience, consecutive, snowball	7
Method of approach	11	How were participants approached? e.g. face-to-face, telephone, mail, email	7
Sample size	12	How many participants were in the study?	7
Non-participation	13	How many people refused to participate or dropped out? Reasons?	7
Setting			
Setting of data collection	14	Where was the data collected? e.g. home, clinic, workplace	7
Presence of non-participants	15	Was anyone else present besides the participants and researchers?	7
Description of sample	16	What are the important characteristics of the sample? e.g. demographic data, date	8
Data collection			
Interview guide	17	Were questions, prompts, guides provided by the authors? Was it pilot tested?	6
Repeat interviews	18	Were repeat inter views carried out? If yes, how many?	7
Audio/visual recording	19	Did the research use audio or visual recording to collect the data?	7
Field notes	20	Were field notes made during and/or after the inter view or focus group?	7
Duration	21	What was the duration of the inter views or focus group?	8
Data saturation	22	Was data saturation discussed?	7
Transcripts returned	23	Were transcripts returned to participants for comment and/or	7

For peer review only

Topic	Item No.	Guide Questions/Description	Reported on Page No.
		correction?	
**Domain 3: analysis and findings**			
Data analysis			
Number of data coders	24	How many data coders coded the data?	7
Description of the coding tree	25	Did authors provide a description of the coding tree?	7
Derivation of themes	26	Were themes identified in advance or derived from the data?	7
Software	27	What software, if applicable, was used to manage the data?	7
Participant checking	28	Did participants provide feedback on the findings?	7
Reporting			
Quotations presented	29	Were participant quotations presented to illustrate the themes/findings? Was each quotation identified? e.g. participant number	9
Data and findings consistent	30	Was there consistency between the data presented and the findings?	9
Clarity of major themes	31	Were major themes clearly presented in the findings?	9
Clarity of minor themes	32	Is there a description of diverse cases or discussion of minor themes?	9

Developed from: Tong A, Sainsbury P, Craig J. Consolidated criteria for reporting qualitative research (COREQ): a 32-item checklist for interviews and focus groups. *International Journal for Quality in Health Care*. 2007. Volume 19, Number 6: pp. 349 – 357

Once you have completed this checklist, please save a copy and upload it as part of your submission. DO NOT include this checklist as part of the main manuscript document. It must be uploaded as a separate file.

For peer review only

BMJ Open

What matters to medical ward patients, and do we measure it?
A qualitative comparison of patient priorities and current practice in quality measurement, on UK NHS medical wards

Journal:	*BMJ Open*
Manuscript ID	bmjopen-2018-024058.R1
Article Type:	Research
Date Submitted by the Author:	19-Sep-2018
Complete List of Authors:	Pannick, Samuel; Charing Cross Hospital, Gastroenterology<br>Archer, Stephanie; Imperial College London, NIHR Imperial Patient Safety Translational Research Centre; Imperial College London,<br>Long, Susannah; Imperial College London, Centre for Patient Safety and Service Quality<br>Husson, Fran; Imperial College, Faculty of Medicine<br>Athanasiou, Thanos; Imperial College London, Surgery and Cancer<br>Sevdalis, Nick; King's College London,
**Primary Subject Heading**:	Health services research
Secondary Subject Heading:	Evidence based practice, Patient-centred medicine, Qualitative research
Keywords:	Quality in health care < HEALTH SERVICES ADMINISTRATION & MANAGEMENT, GENERAL MEDICINE (see Internal Medicine), Organisation of health services < HEALTH SERVICES ADMINISTRATION & MANAGEMENT, Clinical governance < HEALTH SERVICES ADMINISTRATION & MANAGEMENT

SCHOLARONE™ Manuscripts

What matters to medical ward patients, and do we measure it? A qualitative comparison of patient priorities and current practice in quality measurement, on UK NHS medical wards

Samuel Pannick,[1,2] Stephanie Archer,[1] Susannah J Long,[1,2] Fran Husson,[1,2] Thanos Athanasiou,[3] & Nick Sevdalis[4]

[1] NIHR Imperial Patient Safety Translational Research Centre, Imperial College London, UK

[2] Imperial College Healthcare NHS Trust, London, UK

[3] Department of Surgery & Cancer, Imperial College London, UK

[4] Centre for Implementation Science, King's College London, UK

Corresponding author:

Dr Sam Pannick, MA MB BS MRCP PhD

Specialist Registrar in Gastroenterology and Internal Medicine, Department of Gastroenterology, Charing Cross Hospital, Fulham Palace Road, W6 8RF

United Kingdom

sam.pannick@nhs.net; + (44) 203 311 1234

For peer review only

Author information:

Dr Stephanie Archer, Research Fellow, NIHR Imperial Patient Safety Translational Research Centre, Imperial College London.

Dr Susannah J Long, Consultant Geriatrician, Imperial College Healthcare NHS Trust, and Honorary Clinical Senior Lecturer, NIHR Imperial Patient Safety Translational Research Centre, Imperial College London.

Ms Fran Husson, Patient Advocate, Imperial College Healthcare NHS Trust, and Honorary Research Officer, Imperial College London.

Professor Thanos Athanasiou, Consultant Cardiothoracic Surgeon, Imperial College Healthcare NHS Trust, and Professor of Cardiovascular Sciences and Cardiac Surgery, Imperial College London.

Professor Nick Sevdalis, Professor of Implementation Science and Patient Safety, King's College London.

Word count: 3133

Conflicts of interest: This paper represents independent research supported by the National Institute for Health Research (NIHR) Imperial Patient Safety Translational Research Centre, and the Imperial College Healthcare Charity (Grant GG14\1022). NS' research is supported by the NIHR Collaboration for Leadership in Applied Health Research and Care South London at King's College Hospital NHS Foundation Trust. NS is a member of King's Improvement Science, which is part of the NIHR CLAHRC South London and comprises a specialist team of improvement scientists and senior researchers based at King's College London. Its work is funded by King's Health Partners (Guy's and St Thomas' NHS Foundation Trust, King's College Hospital NHS Foundation Trust, King's College London and South London and

For peer review only

Maudsley NHS Foundation Trust), Guy's and St Thomas' Charity, the Maudsley Charity and the Health Foundation. No funding source had any role in the design and conduct of the study; collection, management, analysis or interpretation of the data; or preparation, review or approval of the manuscript. The views expressed are those of the authors and not necessarily those of the NHS, the NIHR or the Department of Health and Social Care.

Contributions:

Study design: SP, SA, TA, FH, NS, SJL. Study implementation and data collection: SA, SP, SJL. Analysis: SA, SP, NS, FH, TA. All authors contributed to, read and approved the final manuscript.

Ethical approval:

Ethical approval was granted by the Westminster Research Ethics Committee (16/LO/0196) and the hospital's joint research compliance office (16SM3129).

Acknowledgements: We are grateful to Drs Louise Hull and Tayana Soukup for their help in facilitating the focus groups. We thank Ms Margaret Turley (carer representative) from the NIHR Imperial PSTRC for her help developing the interview guide.

Data sharing: There are no additional unpublished data.

Key words: healthcare quality; medical ward; patient experience

For peer review only

ABSTRACT

Objectives

To compare the quality metrics selected for public display in NHS medical wards to patients' and carers' expressed quality priorities.

Methods

Qualitative observational assessment of general medical wards and semi-structured interviews.

Setting

UK tertiary NHS (public) hospital

Participants

Fourteen patients and carers on acute medical wards and geriatric wards.

Results

Quality metrics on public display evaluated hand hygiene, hospital-acquired infections, nurse staffing, pressure ulcers, falls, and patient feedback. The intended audience for these metrics was unclear, and the displays gave no indication as to whether performance was improving or worsening. Interviews identified three perceived key components of high quality ward care: communication, staff attitudes, and hygiene. These aligned poorly with the priorities on display. Suboptimal performance reporting had the potential to reduce patients' trust in their medical teams. More philosophically, patients' and carers' ongoing experiences of care would override any other evaluation, and they felt little need for measures relating to previous performance. The display of performance reports only served to emphasise patients' and carers' lack of control in this inpatient setting.

For peer review only

Conclusions

There is a gap between general medical inpatients' care priorities and the aspects of care that are publicly reported. Patients and carers do not act as 'informed choosers' of healthcare in the inpatient setting, and tokenistic quality measurement may have unintended consequences.

For peer review only

STRENGTHS AND LIMITATIONS OF THE STUDY

- Participants included older, frail patients, and those who did not speak English as a first language – demographics often excluded from safety and quality research.
- Our results build on the findings from post-discharge survey studies, free from recall bias.
- Current inpatients are in a vulnerable position and this may have affected some of their interview responses.
- The findings of this single-site study may not be generalisable, although the 'static' performance measures seen at the study site are typical of those reported in other literature.
- We focused on ward displays; other repositories for quality metrics, not in public view, may better approximate patient priorities.

INTRODUCTION

Patient involvement is a priority for the patient safety and healthcare quality movement,[1] but how best to involve patients remains unclear. Policymakers favour the transparent publication of quality metrics (i.e., performance reporting) as a means of engaging patients in their care, framing this engagement as an informed choice of healthcare provider. In the

right context, providing appropriate information can improve patients' and carers' participation in their care, perhaps even improving outcomes.[2] In the UK, this 'informed choice' argument has led to the mandatory display of performance metrics on NHS inpatient wards.[3]

In the acute setting, however, inpatients are unlikely to use performance measures as would typical 'consumers'. These patients are rarely given a choice of provider. Instead, they are assigned to an available ward, or medical team, as determined by organisational capacity. In addition, the debilitation and stress of an acute illness can impede information processing, and intense anxiety can lead to active information avoidance. This may equally affect patients' families or carers, who focus on the immediate health concerns of their loved ones. Even when patients are comfortable accessing complex information at home, they should be treated as 'situationally-impaired' in the hospital environment.[4] Whether inpatients value service-level metrics, and how they relate to them, have not yet been evaluated.

Here, we compare the quality metrics selected for public display in NHS medical wards to patients' and carers' expressed quality priorities. We sought to capture patients' and carers' perceptions of a 'good ward', to better understand their reactions to the quality metrics on display.

METHODS

The study was conducted on general medical wards, which provide the majority of acute inpatient care but struggle for organisational attention or targeted improvement strategies.[5] We assessed ward information displays in two acute medical wards and two geriatric wards at a tertiary NHS (public) hospital in London, with a standardised instrument. This captured the type of performance metrics on public view (e.g., specific hospital-acquired infections or

For peer review only - http://bmjopen.bmj.com/site/about/guidelines.xhtml

pressure ulcers); whether the metrics themselves were clearly defined; whether there was a reference performance benchmark or goal; and how the information was displayed. Free text notes highlighted any adjacent information on the display boards.

Examples were photographed with a digital camera. These images provided insights into the time and priority that the displays were afforded in practice: images are powerful conduits for the feel and texture of environments.[6] Visual materials '*reveal what is hidden in the inner mechanisms of the ordinary*', providing perspective on everday practices.[6] This 'visual sociology', or visual research method, also allows the researcher to reflect on what they encounter in their fieldwork. In doing so, photographic meaning is constructed: one must be aware that photos are not themselves unmediated or unbiased, but dependent on the viewer.[7] Although these documents of record are not undisputed, their value lies in triangulation with other data, in this case the objective categorisation of their contents, and in their interpretation by patients and carers.

The photos were used as prompts in semi-structured interviews with general medical inpatients and their carers at the hospital. Interviews are key tools 'in assessing user views of services and healthcare provision, and in revealing why some care is perceived as poor quality.'[8] The interviews were based on a topic guide, co-developed with patient and carer representatives, exploring care priorities and the concept of a 'good ward' [**Online supplement**]. The topic guide was used flexibly, harnessing broad prompts and follow-up questions, in view of the different roles of the participants (carers and patients) and their varying lengths of hospitalisation.

Ward staff (doctors, nurses, and allied professionals) were asked to suggest patients or carers who would be physically capable of taking part in an interview. Participants were

For peer review only - http://bmjopen.bmj.com/site/about/guidelines.xhtml

aged over 18 years, and able to provide informed consent. Patients were excluded if they were physiologically unstable, had major cognitive or communication difficulties, or did not speak English. The interviews took place at patients' bedsides, as described previously in qualitative work with hospitalised medical patients.[9] Unintentional power relationships and a false therapeutic rapport can develop within sensitive interviews, with implications for data quality.[10,11] To mitigate this, no members of the study team were involved in the participants' clinical care, and this was clearly communicated to the interviewees when they gave their consent to take part in the study. In addition, the interviews were framed as entirely separate from their ongoing clinical care.[11] The interviews were conducted by a specialist registrar in internal medicine and gastroenterology, undertaking a PhD in healthcare quality improvement, with previous experience of qualitative research (SP). Interviews were audiotaped, and then transcribed verbatim. Using NVivo (QSR International, Australia), two researchers trained in qualitative methods (SP – doctor; and SA - psychologist) analysed the transcripts using an inductive (theory-generating) thematic analysis.[12] Each researcher coded the transcripts individually, generating an individual coding frame, which was then discussed and refined between the two coders. The transcripts were coded again, before a group of higher order themes was proposed. A third round of analysis – individually, and then with consensus – confirmed these metathemes and the aggregation of coded transcript fragments within them. The two researchers serially reviewed these results as the interviews were ongoing, and data collection ceased when the study reached saturation, i.e. when no new themes were becoming apparent.

Ethical approval was granted by the Westminster Research Ethics Committee (16/LO/0196) and the hospital's joint research compliance office (16SM3129).

Patient involvement

For peer review only - http://bmjopen.bmj.com/site/about/guidelines.xhtml

The interview topic guide was co-produced with local patient and carer representatives, who in turn canvassed their patient and carer networks for opinions and feedback. The patient representative (FH) co-authored the final manuscript reporting the study's results.

RESULTS

Interview participants

Fourteen people were interviewed (nine patients and five carers). Seven people (four carers) were female. Patients had a median age of 75 (range 57-86), with a median length of stay of five days. 71% of participants spoke English as a first language. 44% (4/9) of patients depended on family or community support, and 33% (3/9) of them had undergone other hospital admissions in the preceding six months. Nine interviews took place on the acute medical wards and five on medicine for the elderly wards. Interviews lasted a median of 23 minutes (range 11-48 minutes).

What performance metrics were on display, and how were they portrayed?

Performance metrics evaluated hand hygiene, hospital-acquired infections (MRSA and C.difficile), nurse staffing, pressure ulcers, falls, and patient feedback [**Online supplement & Table 1**]. The intended audience for these metrics was often unclear: individual display boards contained combinations of messages for patients and staff. Possessive pronouns (our and your) and pronouns (we and you) were used interchangeably, within the same display, to refer to both patients and staff.

Performance measures were displayed with little background information or context. Each metric was displayed as a single, static measure of performance, with no evidence of trends over time. There was no indication of an acceptable benchmark. No patient-actionable information was given for any of the performance measures, other than a suggestion to

For peer review only - http://bmjopen.bmj.com/site/about/guidelines.xhtml

speak to a senior nurse for more information about staffing on the ward. Ward displays about local quality and safety priorities (e.g., 'MRSA compliance') were not explicitly linked to previous performance.

Patient and carer interviews were wide-ranging. For ease of understanding we have aggregated the results into the following sections.

What makes a 'good ward' in the eyes of the patients and their carers?

The interviews identified three key components of high quality ward care: communication, staff attitudes, and hygiene.

1. <u>Communication</u>

Participants felt entirely dependent on staff to keep them abreast of forthcoming investigations and treatments. They valued prompt communication and were keenly aware of its absence. At the same time, they recognised that treatment plans would frequently change, often for reasons outside of their teams' control, and simply held those teams accountable for keeping them updated:

> *'I know it is not always possible that definitive information is available. But as long as you are informed to the ability that they can inform you, you cannot have any gripes about that. If someone says to you, "Look, you may go home tomorrow", I am big enough and ugly enough to know that it may be the day afterwards…' (Patient 3)*

The value of effective, shared communication within the multidisciplinary team was also highlighted. The capacity to speak to one team member, and have that conversation disseminated promptly to the rest of the team, was a key feature of good performance:

For peer review only

'I have found you'll be speaking to one person – and it could be a nurse or a doctor or anybody else – and at the end of the day, everybody knows what I'm talking about... So you can communicate with [just] one person... It's a vital thing.' (Carer 1)

Most comments about information sharing within multidisciplinary teams came from carers, rather than patients. This perhaps reflected the role of carers in the ward environment, where they act both as an information source for professional teams and as advocates for the patients.

2. Staff attitudes

The second element of high quality care was staff attitude. Considerable attention was paid to *how* staff went about their work: staff attentiveness, or 'service', influenced whether patients felt they were on a good ward. Adjectives like 'jolly', 'respectful' and 'helpful', or 'abrupt' and 'wishy-washy', were not so much seen as individual personality attributes, as they were features of work performance:

'[A] good ward is to be helpful to patients, being more human than a machine, you understand?' (Patient 2)

'I think it's the attitude of people [that makes a good ward]. It's the main thing.' (Patient 6)

Thus, the manner of care delivery – rather than the resources available for it – largely defined the care experience. The corollary of this was the potential for a major change

For peer review only

between one shift and the next, even on the same ward. There was a sense, perhaps, that rather than a good or bad ward there were just good shifts or bad shifts:

> *'Where it changes more than anything else is at night, when you have a complete change of staff. Sometimes the night staff that come on are absolutely fantastic, and are very engaged. But sometimes they are entirely the opposite. It is like, "Well we are just here to get you through until the morning, when the people that are looking after you come back."' (Patient 3)*

As well as analysing their own interactions with staff, patients and carers were keen observers of the working relationships between different professionals on the ward. Whether staff seemed appreciative of each other's efforts, or were openly disrespectful to one another, caused patients to wonder how they too were being treated:

> *'[You might think] the more staff, the better the person feels, and that is not how I feel... Everything depends on the lower level[s] of staff we've got working in the ward... and their position [should be] respected by the doctors and the more senior people... They did all [sorts of tasks], and nobody seemed to recognise that they were doing something like that...' (Patient 7)*

> *'Two nurses were having a fight with each other, and that's not very good for the rest of us. And of the course the supervisor was asking them to be quiet, because they were shouting and screaming at each other.' (Patient 4)*

Yet these observations of staff behaviours were quite nuanced. Patients recognised different types of unproductive working relationships, describing over-familiarity (*'almost like a bunch*

For peer review only

of friends working together' – Patient 3), as well as open antagonism. They also made allowances for the general workload on the ward, even excusing displays of inappropriate behaviour:

> *'There's a lot of pressure put on the staff, you know it's understandable. You can see that they're actually very tired people, they needed a good rest, and that's why the whole thing gets on top of them, they're overworked.' (Patient 4)*

3. <u>Hygiene</u>

In a similar vein, patients and carers expressed quite subtle views of why they held hygiene standards to be important. First, good hygiene was *de facto* evidence of a ward that was providing safe care, with little risk of iatrogenic infection. Patients and carers were conscious of the possibility of hospital-acquired infection, understanding it as a major risk associated with inpatient care. Minimising that risk made it possible to focus on the acute medical issues at hand. Second, good hygiene served as a deeper marker of staff pride, diligence and attention to detail, all of which were reassuring:

> *'The cleanliness aspect, I think, is... more important than possibly people realise... It sets out a marker if you like... if the mindset of the ward is, you know, "We are proud of the place that we work in." So it is a fairly good marker of how that ward will actually be.' (Patient 3)*

How did patients and carers perceive the quality metrics on display?

1. <u>Benefits – using infection data for hand hygiene, and understanding staff performance</u>

For peer review only

Patients and carers described some benefits of the quality metrics on display, particularly when it came to infection data. They acknowledged prompts to focus on their own hand hygiene, whilst hoping that staff would do the same. In some cases, a vague familiarity with infection control terminology was helpful:

> *'Because that's in the press [MRSA rates], I suppose people do want to know that, don't they? All of this you read in the papers of people being in hospital - they went in with one thing and they came out with that... You don't want to get worse. They're meant to be making you better.' (Patient 5)*

Real-time information on staffing levels was also potentially helpful, in that it could help set realistic expectations of the care patients might receive:

> *'When I'm getting poor service on a particular day, at least I can see that there might be a good reason for it... I would be more understanding, if I had to wait twice as long for help, if I knew that there was only half the number of staff there should be.' (Patient 8)*

> *'When I saw the amount of staff that you're supposed to have on the ward, there were not half the staff. So the other staff that turned up were constantly busy, running back and forth, and you can see how much stress they were [under]. But they were doing a good job... You can see the nurse who has turned up is doing a really good job.' (Carer 1)*

2. <u>Significant drawbacks – problems in information delivery, prioritisation of personal experience, and unintended consequences</u>

For peer review only

However, patients and carers were largely disparaging about the quality metrics on display. There were numerous problems with information delivery, such as inadequate font size or colour contrast. Yet even with these issues addressed, the information provided was fundamentally inadequate to make a judgement about quality. Patients struggled to see the relevance of a single figure when no trend or benchmark was provided:

> *'Obviously as a member of the public I want the minimum [information], but I have nothing to compare it with. So if you [say], "We've not had one [infection] for three years", I can't compare that with anything. So it doesn't mean anything to me...' (Carer 5)*

> *'That [single figure] doesn't mean anything. That doesn't inform. It could be an increase... but it could be [a] decrease.' (Patient 2)*

More broadly, participants felt little need for measures that related to their wards' previous performance. Their ongoing experiences of care would override any other evaluation. From each individual's perspective, their personal care was the priority, whether or not it reflected a typical standard of care on that ward. In that light, other performance metrics became irrelevant:

> *'I use my own judgement. If I'm satisfied: that's it.' (Patient 9)*

> *'If we want information, we ask for it and we get it. As long as [my relative] is alright and getting looked after, I'm not really bothered about nothing else. If she's getting*

For peer review only

well looked after, the nurses are lovely, their care is great... that's all we are concerned about.' (Carer 3)

As a result, the production of ward quality metrics had some unintended consequences, even going so far as to reduce patients' trust in the whole enterprise. The absence of baseline data in quality displays in particular raised suspicions that poor performance was being concealed.

'Let us face it... you have got your 100% figure there. Would you put up a 20% figure? ... What would you be doing? You would be ruining the confidence of the patients...' (Patient 3)

Patients and carers felt that staff had to have ownership of the quality agenda in hospital: quality metrics were for staff – not patients – to digest. Many interviewees drew comparisons with other settings in which they were consumers: as restaurant diners, or as car purchasers, where their ability to exercise a choice was crucial. Here, however, they had no power to choose, and the display of performance reports only served to emphasise their lack of control:

'It would be great if I'm admitted and I'm given a choice of five wards, and I would say, "Well, how do I know which one's which, which one's best?" My next question would be, "Can you give me the audits of those wards to show which has the highest rating?" and I would go to that... If there's no choice, then it's all academic.' (Patient 8)

For peer review only

'Well, other than clean the wards, there's not a lot we can do is there? What else can you do?' (Carer 4)

The 'Friends and Family Test' question was found to be particularly challenging, given that these patients had no choice in arriving on the ward in the first place, nor could subsequent patients exercise a preference to get there. Indeed, the service pressures on hospital admissions were so well publicised that the idea of choosing a ward seemed faintly ridiculous:

'Would I recommend a ward? How can you recommend a ward?... I mean, that's a daft question, because... they put you in the place you need to be, don't they?' (Patient 5)

DISCUSSION

To our knowledge, this is the first study to compare publicly-displayed performance metrics with patient and carer perceptions of high quality care on UK medical wards. We identified discrepancies between patient- and carer-identified priorities and the quality metrics relating to their care on general medical wards. Patients and carers expressed three core components of high quality general medical care: communication, staff attitudes, and hygiene. These were only partially aligned with the performance measures on display. Specifically, we found process and outcome measures relating to hand hygiene and iatrogenic infection, but none specifically relating to attitudes or communication. Patients and carers acknowledged limited benefits to the display of performance data, but had significant reservations about how it was contextualised. They relied on their own experience of care to judge its quality, above any objective measure of performance. More philosophically, they questioned the purpose of publicly displayed performance data, given

For peer review only

their lack of choice in this setting. In some cases, these reservations actually eroded trust in ward teams' performance.

This study builds on a body of research exploring patient priorities and patient involvement in the acute hospital setting. Boyd surveyed recently-discharged patients, similarly finding that communication, patient-professional interactions, hygiene and the technical delivery of care were their main priorities.[13] Our study suggests that Boyd's findings (which excluded current inpatients) were not unduly affected by recall bias. Nonetheless, hospitalised patients remain relatively indifferent to service-level performance and change.[14] We suggest an explanation for this: current inpatients are unable to exercise informed choices about their ward, nor are they able to directly use information to improve performance. They are therefore excluded from the two key pathways by which performance measurement may lead to quality improvement.[15]

Our findings question the mandatory collection, and display, of performance data that do not align with patient priorities. These data collection exercises have considerable opportunity costs. We note the recent call for the abolition of the mandatory 'Friends and family test', one of the performance indicators we found on display, which has been criticised on similar lines.[16] These data sets are expensive to maintain*, "at best tolerated, often ignored, and sometimes ridiculed"*.[16] The resulting tokenistic display of performance data erodes patients' trust in the system that organises and governs their care. It can also be corrosive for staff morale, both at the frontline and at board level.[17,18] This tokenism is perpetuated by a dearth of resources for implementing meaningful improvement.[1,19] A credible, co-produced, quality framework for acute medical inpatients is urgently required, with outcomes that are sensitive to the work[20] and structures[21] of inpatient care. Co-

For peer review only - http://bmjopen.bmj.com/site/about/guidelines.xhtml

produced quality standards should capitalise on the active contributions of patients and carers, rather than depicting them as ‘informed choosers’ of healthcare provision.

Study limitations include a relatively small sample from a single site. We collected demographic data for patients but not their carers. Nonetheless, the group of interviewees included demographics often excluded from safety and quality research: older, frail patients, and those who do not speak English as a first language.[22] The study reached data saturation, with no new themes emerging as the final interviews took place. Ward displays at this site were also typical for regional practice. ‘Static’ performance measures, as seen here, are widespread, and even the performance data presented at healthcare board level rarely depicts the role of chance in the formation of data patterns.[23,24] Finally, other repositories for quality metrics, beyond those ward displays analysed here, may better approximate patient priorities. However, they typically use composites of the data we found,[25] or are aggregated to the hospital level, with no ward-level interpretation.[26,27]

CONCLUSION

There is a gap between general medical inpatients’ care priorities and the aspects of care that are publicly reported. Tokenistic quality measurement may have unintended consequences, eroding patients’ trust in ward teams.

REFERENCES

1. Lawton R, O'Hara JK, Sheard L, et al. Can patient involvement improve patient safety? A cluster randomised control trial of the Patient Reporting and Action for a Safe Environment (PRASE) intervention. BMJ Qual Saf 2017;**26**(8):622-31.
2. Shaller D, Kanouse DE, Schlesinger M. Context-based strategies for engaging consumers with public reports about health care providers. Med Care Res Rev 2014;**71**(5 Suppl):17S-37S.
3. Department of Health. Hospital 'friends and family test' announced. Available at https://www.gov.uk/government/news/hospital-friends-and-family-test-announced. Accessed 11/12/2017.

4. Morris D, Karlson A. Dynamic Accessibility Requirements for Hospital Patients. SIGCHI Conference on Human Factors in Computing Systems. Vancouver, Canada: ACM Press, 2011.
5. Pannick S, Wachter RM, Vincent C, et al. Rethinking medical ward quality. BMJ 2016;**355**:i5417.
6. Rose G. On the relation between 'visual research methods' and contemporary visual culture. The Sociological Review 2014;**62**(1):24-46.
7. Schwartz D. Visual ethnography: Using photography in qualitative research. Qualitative Sociology 1989;**12**(2):119-54.
8. Pope C, van Royen P, Baker R. Qualitative methods in research on healthcare quality. Quality and Safety in Health Care 2002;**11**(2):148-52.
9. Tobiano G, Bucknall T, Marshall A, et al. Patients' perceptions of participation in nursing care on medical wards. Scand J Caring Sci 2016;**30**(2):260-70.
10. Sivell S, Prout H, Baillie J, et al. Conducting qualitative research interviews with palliative and end of life care patients in the home setting; reflections and recommendations from an academic palliative care research centre. BMJ Supportive & Palliative Care. 2015;**5**(1):117-17.
11. Sivell S, Prout H, Hopewell-Kelly N, et al. Considerations and recommendations for conducting qualitative research interviews with palliative and end-of-life care patients in the home setting: a consensus paper. BMJ Supportive & Palliative Care 2015;10.1136/bmjspcare-2015-000892.
12. Braun V, Clarke V. Using thematic analysis in psychology. Qualitative Research in Psychology 2006;**3**(2):77-101.
13. Boyd J. The 2006 inpatients importantance study. Oxford: Picker Institute, 2007. http://www.nhssurveys.org/Filestore/documents/Findings_and_development_of_the_2006_Inpatients_Importance_study_final.pdf.
14. Wray CM, Farnan JM, Arora VM, et al. A qualitative analysis of patients' experience with hospitalist service handovers. J Hosp Med 2016;10.1002/jhm.2608.
15. Berwick DM, James B, Coye MJ. Connections between quality measurement and improvement. Med Care 2003;**41**(1 Suppl):I30-8.
16. Robert G, Cornwell J, Black N. Friends and family test should no longer be mandatory. BMJ 2018;**360**:k367.
17. NHS England. Review of the Friends and Family Test. 21st July 2014. Available at https://www.england.nhs.uk/wp-content/uploads/2014/07/fft-rev1.pdf. Accessed 16/10/2017.
18. Raleigh V, Thompson J, Jabbal J, et al. Patients' experience of using hospital services. London: The King's Fund, 2015. http://www.pickereurope.org/wp-content/uploads/2015/12/Patients-experience-Kings-Fund-Dec-2015.pdf.
19. Sheard L, Marsh C, O'Hara J, et al. The Patient Feedback Response Framework - Understanding why UK hospital staff find it difficult to make improvements based on patient feedback: A qualitative study. Soc Sci Med 2017;**178**:19-27.
20. Pannick S, Davis R, Ashrafian H, et al. Effects of Interdisciplinary Team Care Interventions on General Medical Wards: A Systematic Review. JAMA Intern Med 2015;**175**(8):1288-98.
21. Dumitrascu AG, Burton MC, Dawson NL, et al. Patient portal use and hospital outcomes. Journal of the American Medical Informatics Association 2018;**25**(4):447-53.
22. O'Hara JK, Lawton RJ. At a crossroads? Key challenges and future opportunities for patient involvement in patient safety. BMJ Qual Saf 2016;10.1136/bmjqs-2016-005476.

For peer review only

23. Anhoj J, Hellesoe AB. The problem with red, amber, green: the need to avoid distraction by random variation in organisational performance measures. BMJ Qual Saf 2016;10.1136/bmjqs-2015-004951.
24. Schmidtke KA, Poots AJ, Carpio J, et al. Considering chance in quality and safety performance measures: an analysis of performance reports by boards in English NHS trusts. BMJ Qual Saf 2017;**26**(1):61-69.
25. NHS Quality Observatory. NHS Safety Thermometer. Available at https://www.safetythermometer.nhs.uk/index.php?option=com_content&view=article&id=1&Itemid=101. Accessed 24th June 2016.
26. Care Quality Commission. NHS Surveys: focused on patients' experience. Available at http://www.nhssurveys.org/. Accessed 23rd June 2016.
27. Centers for Medicare and Medicaid Services. HCAHPS Fact Sheet. June 2015. Baltimore, MD. Available at http://www.hcahpsonline.org/facts.aspx. Accessed 23rd June 2016.
28. NHS England. Friends and Family Test: Guidance. 2014. Publications Gateway Ref No. 01787.

For peer review only

Table 1: Performance indicators available in public ward areas

Performance indicator	*Display choice*	*Display format*
Hand hygiene	Percentage in last audit	Most recent result only; numerator and denominator definitions not provided.
Hospital-acquired infections	Date of last recorded event	Most recent result only
Pressure ulcers	Date of last recorded event	Most recent result only
Falls	Date of last recorded event	Most recent result only
Nurse staffing	Numbers of staff required for the shift *vs* those actually on duty, for staff nurses and health care assistants	Most recent result only; explanation of staff responsibilities
Patient feedback	'Friends and Family Test' star rating; percentage of patients who would recommend the ward*	Most recent result only; examples of patients' comments; no explanation of star rating system

*The 'Friends and Family Test' asks *"How likely are you to recommend our service to friends and family if they needed similar care or treatment?"*[28]

For peer review only

TOPIC GUIDE: Current patient / carer for current patient

1. Let's find out about you.

Age
Employment
Education – school / university / postgraduate
Social support structures & marital status
Ethnicity

2. Why are you in hospital now?

Current diagnosis
Other conditions
Approximate length of stay to date
< 1 day
1 – 5 days
5 – 10 days
> 10 days

3. How many times have you been admitted to hospital in the last 6 months?

1-5
5-10
>10

4. Do you always come to this hospital or have you been admitted to other local hospitals?

5. How do you know if you're on a good ward? What is a 'good ward' to you?

Environment
- Clean
- Quiet
- Toilet and shower are available when required
- Meal timeliness, warmth
- Help available when requested
- Staff are responsiveness to my needs / my family's needs

Welcome
- My arrival is expected
- Staff introduce themselves
- Staff make me feel I will be well looked after; show a caring attitude; and don't rush me

Communication and use of personal information
- Accurate knowledge of previous medical history / current diagnosis / current investigations / discharge plan / medication reconciliation
- Quality of communication / teamwork

Discharge preparation

For peer review only

Friends' / families' recommendations

Ward information boards / quality and safety boards

Ward information leaflets / other printed materials.

Ward information displays / electronic screens

6. **If you had to decide whether a ward was good or not, what information would you need to make that decision?**

7. **Have you noticed any of the information the ward displays about itself? What do you think of the information you've seen?**

Friends and family test results
Safety cross
Shift-by-shift staffing
Falls
Pressure ulcers
Safety thermometer / harm-free care
Venous thromboembolism prophylaxis
Hand hygiene compliance
Hospital-acquired infections
Infection rates
Incident reporting

8. **What would you like to know about how your ward is performing?**

Hand hygiene compliance
Staffing levels
Friends and family results
Hospital-acquired infections
Pressure Ulcers
Falls
Venous thromboembolism prophylaxis
Complaints
Compliments
Length of stay
Mortality
Readmission rate
Safety climate

9. **How should your ward make that information available to you and your family??**

Ward displays
Leaflets
Smartphone / other device
Webpage

For peer review only

10. Preference for information seeking

Information-seeking sub-scale

	Disagree strongly	Disagree slightly	Neutral	Agree slightly	Agree strongly
As you become sicker you should be told more and more about your illness					
You should understand completely what is happening inside your body as a result of your illness					
Even if the news is bad, you should be well informed					
Your doctor should explain the purpose of your laboratory tests					
It is important for you to know all the side effects of your medication					
Information about your illness is as important to you as treatment					
When there is more than one method to treat a problem, you should be told about each one					

11. Have you previously had to complain about care or healthcare staff, nurses or doctors? What made you complain? How? PALS / informally / in writing?

For peer review only

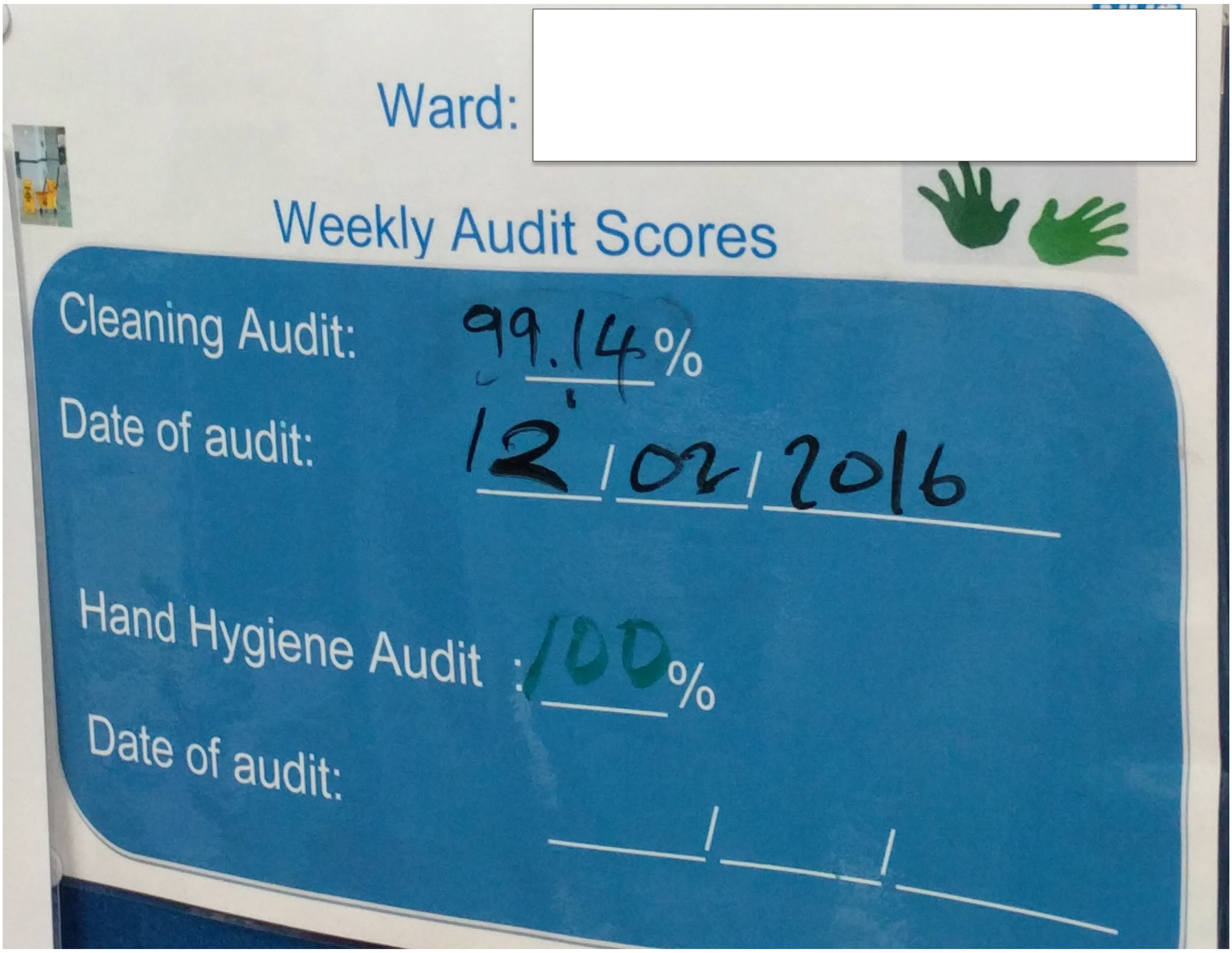
Ward:
Weekly Audit Scores
Cleaning Audit: 99.14%
Date of audit: 12/02/2016
Hand Hygiene Audit : 100%
Date of audit:

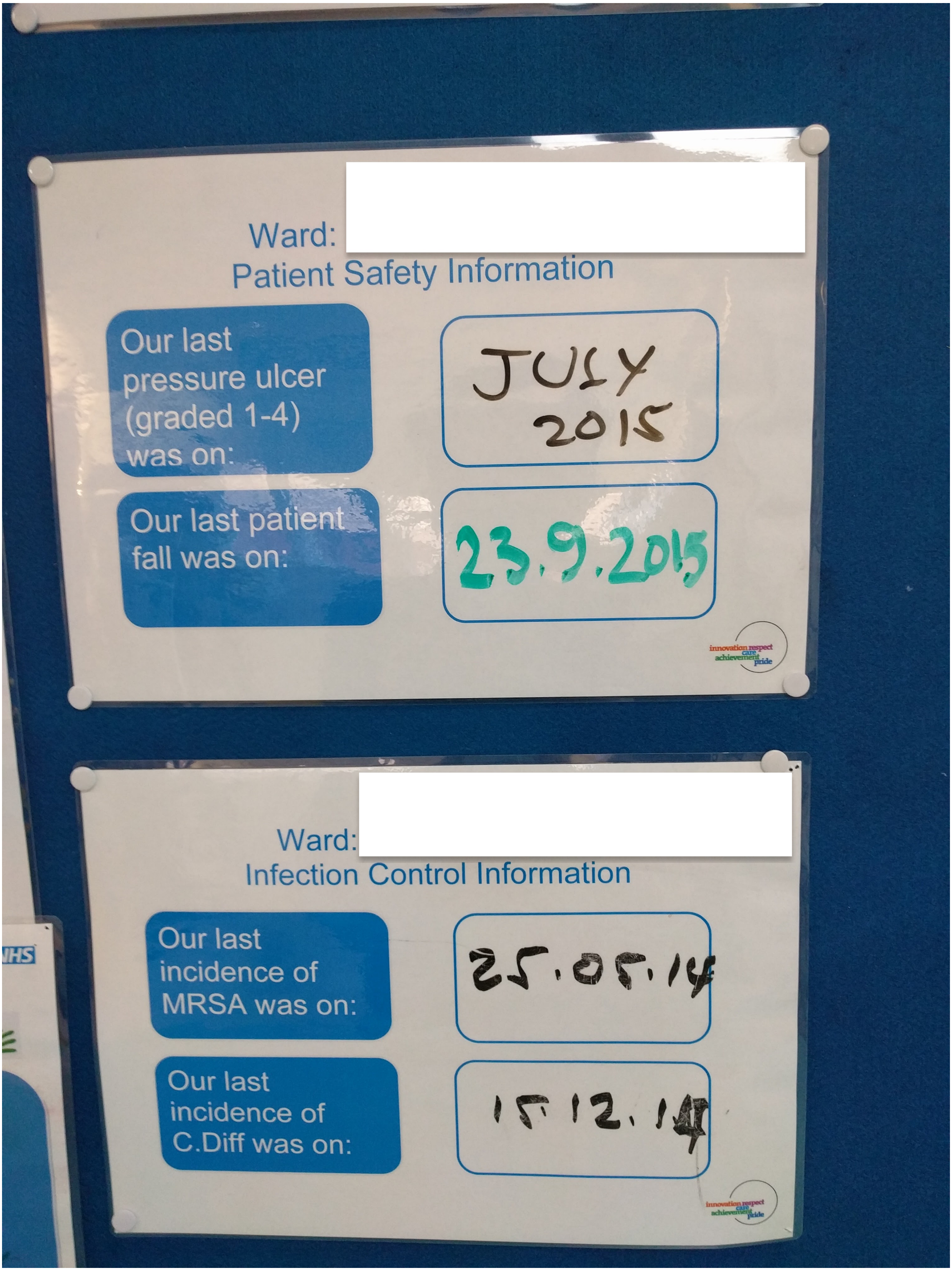
Ward:
Patient Safety Information
Our last pressure ulcer (graded 1-4) was on:
JULY 2015
Our last patient fall was on:
23.9.2015
Ward:
Infection Control Information
Our last incidence of MRSA was on:
25.05.14
Our last incidence of C.Diff was on:
15.12.14

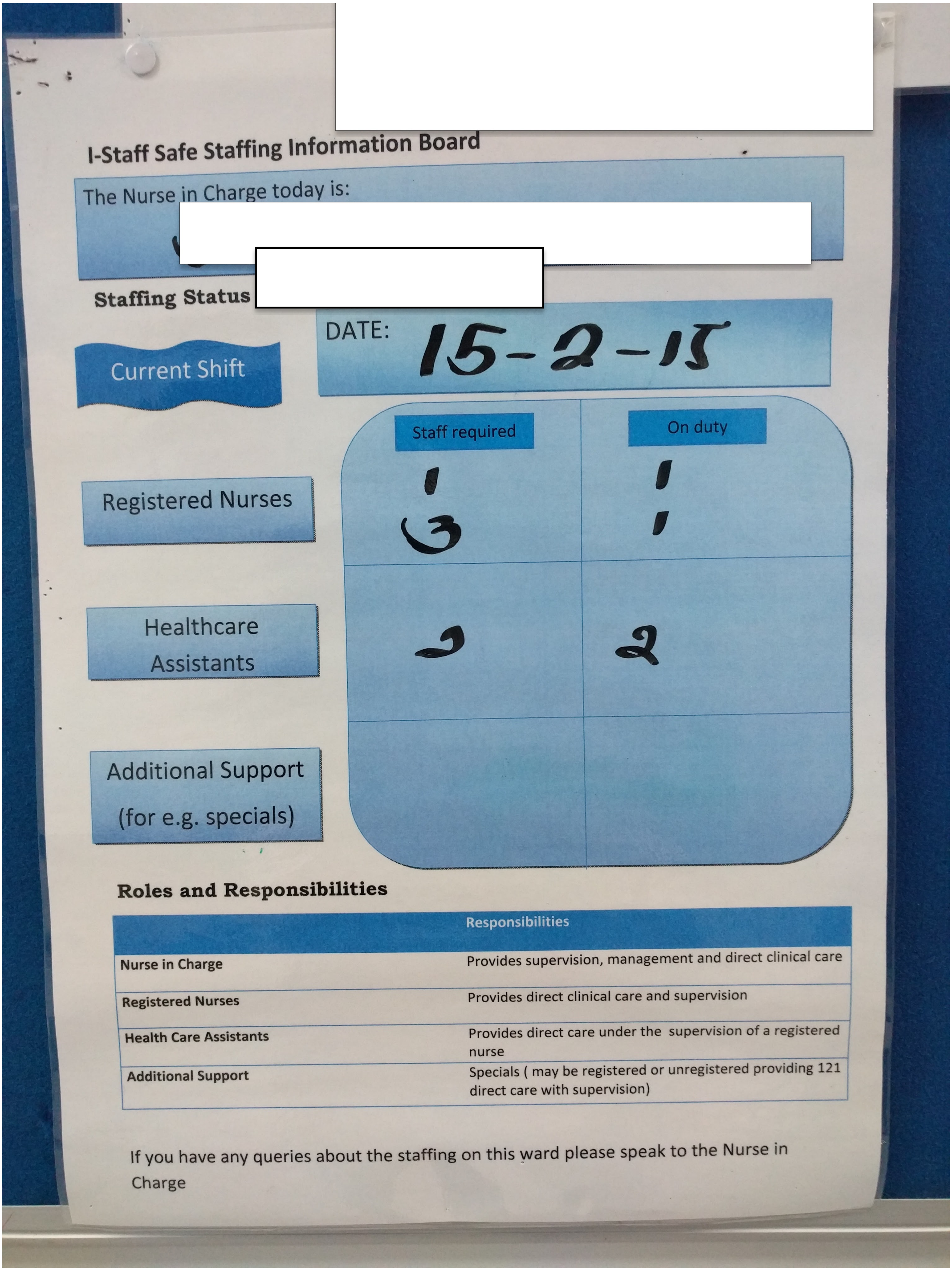
I-Staff Safe Staffing Information Board
The Nurse in Charge today is:
Staffing Status
DATE: 15-2-15
Current Shift
Staff required
On duty
Registered Nurses
1
3
1
1
Healthcare Assistants
2
2
Additional Support (for e.g. specials)
Roles and Responsibilities
Responsibilities
Nurse in Charge
Provides supervision, management and direct clinical care
Registered Nurses
Provides direct clinical care and supervision
Health Care Assistants
Provides direct care under the supervision of a registered nurse
Additional Support
Specials (may be registered or unregistered providing 121 direct care with supervision)
If you have any queries about the staffing on this ward please speak to the Nurse in Charge

COREQ (COnsolidated criteria for REporting Qualitative research) Checklist

A checklist of items that should be included in reports of qualitative research. You must report the page number in your manuscript where you consider each of the items listed in this checklist. If you have not included this information, either revise your manuscript accordingly before submitting or note N/A.

Topic	Item No.	Guide Questions/Description	Reported on Page No.
**Domain 1: Research team and reflexivity**			
Personal characteristics			
Interviewer/facilitator	1	Which author/s conducted the interview or focus group?	7
Credentials	2	What were the researcher's credentials? E.g. PhD, MD	7
Occupation	3	What was their occupation at the time of the study?	7
Gender	4	Was the researcher male or female?	7
Experience and training	5	What experience or training did the researcher have?	7
Relationship with participants			
Relationship established	6	Was a relationship established prior to study commencement?	7
Participant knowledge of the interviewer	7	What did the participants know about the researcher? e.g. personal goals, reasons for doing the research	7
Interviewer characteristics	8	What characteristics were reported about the inter viewer/facilitator? e.g. Bias, assumptions, reasons and interests in the research topic	7
**Domain 2: Study design**			
Theoretical framework			
Methodological orientation and Theory	9	What methodological orientation was stated to underpin the study? e.g. grounded theory, discourse analysis, ethnography, phenomenology, content analysis	7
Participant selection			
Sampling	10	How were participants selected? e.g. purposive, convenience, consecutive, snowball	7
Method of approach	11	How were participants approached? e.g. face-to-face, telephone, mail, email	7
Sample size	12	How many participants were in the study?	7
Non-participation	13	How many people refused to participate or dropped out? Reasons?	7
Setting			
Setting of data collection	14	Where was the data collected? e.g. home, clinic, workplace	7
Presence of non-participants	15	Was anyone else present besides the participants and researchers?	7
Description of sample	16	What are the important characteristics of the sample? e.g. demographic data, date	8
Data collection			
Interview guide	17	Were questions, prompts, guides provided by the authors? Was it pilot tested?	6
Repeat interviews	18	Were repeat inter views carried out? If yes, how many?	7
Audio/visual recording	19	Did the research use audio or visual recording to collect the data?	7
Field notes	20	Were field notes made during and/or after the inter view or focus group?	7
Duration	21	What was the duration of the inter views or focus group?	8
Data saturation	22	Was data saturation discussed?	7
Transcripts returned	23	Were transcripts returned to participants for comment and/or	7

For peer review only

Topic	Item No.	Guide Questions/Description	Reported on Page No.
		correction?	
**Domain 3: analysis and findings**			
Data analysis			
Number of data coders	24	How many data coders coded the data?	7
Description of the coding tree	25	Did authors provide a description of the coding tree?	7
Derivation of themes	26	Were themes identified in advance or derived from the data?	7
Software	27	What software, if applicable, was used to manage the data?	7
Participant checking	28	Did participants provide feedback on the findings?	7
Reporting			
Quotations presented	29	Were participant quotations presented to illustrate the themes/findings? Was each quotation identified? e.g. participant number	9
Data and findings consistent	30	Was there consistency between the data presented and the findings?	9
Clarity of major themes	31	Were major themes clearly presented in the findings?	9
Clarity of minor themes	32	Is there a description of diverse cases or discussion of minor themes?	9

Developed from: Tong A, Sainsbury P, Craig J. Consolidated criteria for reporting qualitative research (COREQ): a 32-item checklist for interviews and focus groups. *International Journal for Quality in Health Care*. 2007. Volume 19, Number 6: pp. 349 – 357

Once you have completed this checklist, please save a copy and upload it as part of your submission. DO NOT include this checklist as part of the main manuscript document. It must be uploaded as a separate file.

For peer review only

BMJ Open

What matters to medical ward patients, and do we measure it?
A qualitative comparison of patient priorities and current practice in quality measurement, on UK NHS medical wards

Journal:	*BMJ Open*
Manuscript ID	bmjopen-2018-024058.R2
Article Type:	Research
Date Submitted by the Author:	18-Dec-2018
Complete List of Authors:	Pannick, Samuel; Charing Cross Hospital, Gastroenterology<br>Archer, Stephanie; Imperial College London, NIHR Imperial Patient Safety Translational Research Centre; Imperial College London,<br>Long, Susannah; Imperial College London, Centre for Patient Safety and Service Quality<br>Husson, Fran; Imperial College, Faculty of Medicine<br>Athanasiou, Thanos; Imperial College London, Surgery and Cancer<br>Sevdalis, Nick; King's College London,
**Primary Subject Heading**:	Health services research
Secondary Subject Heading:	Evidence based practice, Patient-centred medicine, Qualitative research
Keywords:	Quality in health care < HEALTH SERVICES ADMINISTRATION & MANAGEMENT, GENERAL MEDICINE (see Internal Medicine), Organisation of health services < HEALTH SERVICES ADMINISTRATION & MANAGEMENT, Clinical governance < HEALTH SERVICES ADMINISTRATION & MANAGEMENT

SCHOLARONE™
Manuscripts

What matters to medical ward patients, and do we measure it?

A qualitative comparison of patient priorities and current practice in quality measurement, on UK NHS medical wards

Samuel Pannick,[1,2] Stephanie Archer,[1] Susannah J Long,[1,2] Fran Husson,[1,2] Thanos Athanasiou,[3] & Nick Sevdalis[4]

[1] NIHR Imperial Patient Safety Translational Research Centre, Imperial College London, UK

[2] Imperial College Healthcare NHS Trust, London, UK

[3] Department of Surgery & Cancer, Imperial College London, UK

[4] Centre for Implementation Science, King's College London, UK

Corresponding author:

Dr Sam Pannick, MA MB BS MRCP PhD

Specialist Registrar in Gastroenterology and Internal Medicine, Department of Gastroenterology, Charing Cross Hospital, Fulham Palace Road, W6 8RF

United Kingdom

sam.pannick@nhs.net; + (44) 203 311 1234

For peer review only

Author information:

Dr Stephanie Archer, Research Fellow, NIHR Imperial Patient Safety Translational Research Centre, Imperial College London.

Dr Susannah J Long, Consultant Geriatrician, Imperial College Healthcare NHS Trust, and Honorary Clinical Senior Lecturer, NIHR Imperial Patient Safety Translational Research Centre, Imperial College London.

Ms Fran Husson, Patient Advocate, Imperial College Healthcare NHS Trust, and Honorary Research Officer, Imperial College London.

Professor Thanos Athanasiou, Consultant Cardiothoracic Surgeon, Imperial College Healthcare NHS Trust, and Professor of Cardiovascular Sciences and Cardiac Surgery, Imperial College London.

Professor Nick Sevdalis, Professor of Implementation Science and Patient Safety, King's College London.

Conflicts of interest: This paper represents independent research supported by the National Institute for Health Research (NIHR) Imperial Patient Safety Translational Research Centre, and the Imperial College Healthcare Charity (Grant GG14\1022). NS' research is supported by the NIHR Collaboration for Leadership in Applied Health Research and Care South London at King's College Hospital NHS Foundation Trust. NS is a member of King's Improvement Science, which is part of the NIHR CLAHRC South London and comprises a specialist team of improvement scientists and senior researchers based at King's College London. Its work is funded by King's Health Partners (Guy's and St Thomas' NHS Foundation Trust, King's College Hospital NHS Foundation Trust, King's College London and South London and Maudsley NHS Foundation Trust), Guy's and St Thomas' Charity, the Maudsley Charity and the Health Foundation. No funding source had any role in the design and conduct of the

For peer review only

For peer review only - http://bmjopen.bmj.com/site/about/guidelines.xhtml

study; collection, management, analysis or interpretation of the data; or preparation, review or approval of the manuscript. The views expressed are those of the authors and not necessarily those of the NHS, the NIHR or the Department of Health and Social Care.

Contributions:

Study design: SP, SA, TA, FH, NS, SJL. Study implementation and data collection: SA, SP, SJL. Analysis: SA, SP, NS, FH, TA. All authors contributed to, read and approved the final manuscript.

Ethical approval:

Ethical approval was granted by the Westminster Research Ethics Committee (16/LO/0196) and the hospital's joint research compliance office (16SM3129).

Acknowledgements: We are grateful to Drs Louise Hull and Tayana Soukup for their help in facilitating the focus groups. We thank Ms Margaret Turley (carer representative) from the NIHR Imperial PSTRC for her help developing the interview guide.

Data sharing: There are no additional unpublished data.

Key words: healthcare quality; medical ward; patient experience

For peer review only

ABSTRACT

Objectives

To compare the quality metrics selected for public display in NHS medical wards to patients' and carers' expressed quality priorities.

Methods

Multi-modal qualitative evaluation of general medical wards and semi-structured interviews.

Setting

UK tertiary NHS (public) hospital

Participants

Fourteen patients and carers on acute medical wards and geriatric wards.

Results

Quality metrics on public display evaluated hand hygiene, hospital-acquired infections, nurse staffing, pressure ulcers, falls, and patient feedback. The intended audience for these metrics was unclear, and the displays gave no indication as to whether performance was improving or worsening. Interviews identified three perceived key components of high quality ward care: communication, staff attitudes, and hygiene. These aligned poorly with the priorities on display. Suboptimal performance reporting had the potential to reduce patients' trust in their medical teams. More philosophically, patients' and carers' ongoing experiences of care would override any other evaluation, and they felt little need for measures relating to previous performance. The display of performance reports only served to emphasise patients' and carers' lack of control in this inpatient setting.

For peer review only - http://bmjopen.bmj.com/site/about/guidelines.xhtml

Conclusions

There is a gap between general medical inpatients' care priorities and the aspects of care that are publicly reported. Patients and carers do not act as 'informed choosers' of healthcare in the inpatient setting, and tokenistic quality measurement may have unintended consequences.

STRENGTHS AND LIMITATIONS OF THE STUDY

- Participants included older, frail patients, and those who did not speak English as a first language – demographics often excluded from safety and quality research.
- Our results build on the findings from post-discharge survey studies, free from recall bias.
- Current inpatients are in a vulnerable position and this may have affected some of their interview responses.
- The findings of this single-site study may not be generalisable, although the 'static' performance measures seen at the study site are typical of those reported in other literature.
- We focused on ward displays; other repositories for quality metrics, not in public view, may better approximate patient priorities.

INTRODUCTION

Patient involvement is a priority for the patient safety and healthcare quality movement,[1] but how best to involve patients remains unclear. Policymakers favour the transparent publication of quality metrics (i.e., performance reporting) as a means of engaging patients in their care, framing this engagement as an informed choice of healthcare provider. In the right context, providing appropriate information can improve patients' and carers'

For peer review only

participation in their care, perhaps even improving outcomes.[2] In the UK, this ‘informed choice’ argument has led to the mandatory display of performance metrics on NHS inpatient wards.[3]

In the acute setting, however, inpatients are unlikely to use performance measures as would typical ‘consumers’. These patients are rarely given a choice of provider. Instead, they are assigned to an available ward, or medical team, as determined by organisational capacity. In addition, the debilitation and stress of an acute illness can impede information processing, and intense anxiety can lead to active information avoidance. This may equally affect patients’ families or carers, who focus on the immediate health concerns of their loved ones. Even when patients are comfortable accessing complex information at home, they should be treated as ‘situationally-impaired’ in the hospital environment.[4] Whether inpatients value service-level metrics, and how they relate to them, have not yet been evaluated.

Here, we compare the quality metrics selected for public display in NHS medical wards to patients’ and carers’ expressed quality priorities. We sought to capture patients’ and carers’ perceptions of a ‘good ward’, to better understand their reactions to the quality metrics on display.

METHODS

The study was conducted on general medical wards, which provide the majority of acute inpatient care but struggle for organisational attention or targeted improvement strategies.[5] We assessed ward information displays in two acute medical wards and two geriatric wards at a tertiary NHS (public) hospital in London, with a proforma. This captured the type of performance metrics on public view (e.g., specific hospital-acquired infections or pressure ulcers); whether the metrics themselves were clearly defined; whether there was a

For peer review only - http://bmjopen.bmj.com/site/about/guidelines.xhtml

reference performance benchmark or goal; and how the information was displayed. Free text notes highlighted any adjacent information on the display boards.

Examples were photographed with a digital camera. These images provided insights into the time and priority that the displays were afforded in practice: images are powerful conduits for the feel and texture of environments.[6] Visual materials '*reveal what is hidden in the inner mechanisms of the ordinary'*, providing perspective on everyday practices.[6] This 'visual sociology', or visual research method, also allows the researcher to reflect on what they encounter in their fieldwork. In doing so, photographic meaning is constructed: one must be aware that photos are not themselves unmediated or unbiased, but dependent on the viewer.[7] Although these documents of record are not undisputed, their value lies in triangulation with other data, in this case the objective categorisation of their contents, and in their interpretation by patients and carers.

The photos were used as prompts in semi-structured interviews with general medical inpatients and their carers at the hospital. Interviews are key tools 'in assessing user views of services and healthcare provision, and in revealing why some care is perceived as poor quality.'[8] The interviews were based on a topic guide, co-developed with patient and carer representatives, exploring care priorities and the concept of a 'good ward' [**Online supplement**]. The topic guide was used flexibly, harnessing broad prompts and follow-up questions, in view of the different roles of the participants (carers and patients) and their varying lengths of hospitalisation.

Ward staff (doctors, nurses, and allied professionals) were asked to suggest patients or carers who would be physically capable of taking part in an interview. Participants were aged over 18 years, and able to provide informed consent. Patients were excluded if they

For peer review only - http://bmjopen.bmj.com/site/about/guidelines.xhtml

were physiologically unstable, had major cognitive or communication difficulties, or did not speak English. The interviews took place at patients' bedsides, as described previously in qualitative work with hospitalised medical patients.[9] Unintentional power relationships and a false therapeutic rapport can develop within sensitive interviews, with implications for data quality.[10,11] To mitigate this, no members of the study team were involved in the participants' clinical care, and this was clearly communicated to the interviewees when they gave their consent to take part in the study. In addition, the interviews were framed as entirely separate from their ongoing clinical care.[11] The interviews were conducted by a specialist registrar in internal medicine and gastroenterology, undertaking a PhD in healthcare quality improvement, with previous experience of qualitative research (SP). Interviews were audiotaped, and then transcribed verbatim. Using NVivo (QSR International, Australia), two researchers trained in qualitative methods (SP – doctor; and SA - psychologist) analysed the transcripts using an inductive (theory-generating) thematic analysis.[12] Each researcher coded the transcripts individually, generating an individual coding frame, which was then discussed and refined between the two coders. The transcripts were coded again, before a group of higher order themes was proposed. A third round of analysis – individually, and then with consensus – confirmed these metathemes and the aggregation of coded transcript fragments within them. The two researchers serially reviewed these results as the interviews were ongoing, and data collection ceased when the study reached saturation, i.e. when no new themes were becoming apparent.

Ethical approval was granted by the Westminster Research Ethics Committee (16/LO/0196) and the hospital's joint research compliance office (16SM3129).

Patient involvement

For peer review only

The interview topic guide was co-produced with local patient and carer representatives, who in turn canvassed their patient and carer networks for opinions and feedback. The patient representative (FH) co-authored the final manuscript reporting the study's results.

RESULTS

Interview participants

Fourteen people were interviewed (nine patients and five carers). Seven people (four carers) were female. Patients had a median age of 75 (range 57-86), with a median length of stay of five days. 71% of participants spoke English as a first language. 44% (4/9) of patients depended on family or community support, and 33% (3/9) of them had undergone other hospital admissions in the preceding six months. Nine interviews took place on the acute medical wards and five on medicine for the elderly wards. Interviews lasted a median of 23 minutes (range 11-48 minutes).

What performance metrics were on display, and how were they portrayed?

Performance metrics evaluated hand hygiene, hospital-acquired infections (MRSA and C.difficile), nurse staffing, pressure ulcers, falls, and patient feedback [**Online supplement & Table 1**]. The intended audience for these metrics was often unclear: individual display boards contained combinations of messages for patients and staff. Possessive pronouns (our and your) and pronouns (we and you) were used interchangeably, within the same display, to refer to both patients and staff.

Performance measures were displayed with little background information or context. Each metric was displayed as a single, static measure of performance, with no evidence of trends over time. There was no indication of an acceptable benchmark. No patient-actionable information was given for any of the performance measures, other than a suggestion to

For peer review only - http://bmjopen.bmj.com/site/about/guidelines.xhtml

speak to a senior nurse for more information about staffing on the ward. Ward displays about local quality and safety priorities (e.g., 'MRSA compliance') were not explicitly linked to previous performance.

Patient and carer interviews were wide-ranging. For ease of understanding we have aggregated the results into the following sections.

What makes a 'good ward' in the eyes of the patients and their carers?

The interviews identified three key components of high quality ward care: communication, staff attitudes, and hygiene.

1. Communication

Participants felt entirely dependent on staff to keep them abreast of forthcoming investigations and treatments. They valued prompt communication and were keenly aware of its absence. At the same time, they recognised that treatment plans would frequently change, often for reasons outside of their teams' control, and simply held those teams accountable for keeping them updated:

> *'I know it is not always possible that definitive information is available. But as long as you are informed to the ability that they can inform you, you cannot have any gripes about that. If someone says to you, "Look, you may go home tomorrow", I am big enough and ugly enough to know that it may be the day afterwards…' (Patient 3)*

The value of effective, shared communication within the multidisciplinary team was also highlighted. The capacity to speak to one team member, and have that conversation disseminated promptly to the rest of the team, was a key feature of good performance:

For peer review only - http://bmjopen.bmj.com/site/about/guidelines.xhtml

'I have found you'll be speaking to one person – and it could be a nurse or a doctor or anybody else – and at the end of the day, everybody knows what I'm talking about... So you can communicate with [just] one person... It's a vital thing.' (Carer 1)

Most comments about information sharing within multidisciplinary teams came from carers, rather than patients. This perhaps reflected the role of carers in the ward environment, where they act both as an information source for professional teams and as advocates for the patients.

2. Staff attitudes

The second element of high quality care was staff attitude. Considerable attention was paid to *how* staff went about their work: staff attentiveness, or 'service', influenced whether patients felt they were on a good ward. Adjectives like 'jolly', 'respectful' and 'helpful', or 'abrupt' and 'wishy-washy', were not so much seen as individual personality attributes, as they were features of work performance:

'[A] good ward is to be helpful to patients, being more human than a machine, you understand?' (Patient 2)

'I think it's the attitude of people [that makes a good ward]. It's the main thing.' (Patient 6)

Thus, the manner of care delivery – rather than the resources available for it – largely defined the care experience. The corollary of this was the potential for a major change

For peer review only

between one shift and the next, even on the same ward. There was a sense, perhaps, that rather than a good or bad ward there were just good shifts or bad shifts:

> *'Where it changes more than anything else is at night, when you have a complete change of staff. Sometimes the night staff that come on are absolutely fantastic, and are very engaged. But sometimes they are entirely the opposite. It is like, "Well we are just here to get you through until the morning, when the people that are looking after you come back."' (Patient 3)*

As well as analysing their own interactions with staff, patients and carers were keen observers of the working relationships between different professionals on the ward. Whether staff seemed appreciative of each other's efforts, or were openly disrespectful to one another, caused patients to wonder how they too were being treated:

> *'[You might think] the more staff, the better the person feels, and that is not how I feel... Everything depends on the lower level[s] of staff we've got working in the ward... and their position [should be] respected by the doctors and the more senior people... They did all [sorts of tasks], and nobody seemed to recognise that they were doing something like that...' (Patient 7)*

> *'Two nurses were having a fight with each other, and that's not very good for the rest of us. And of the course the supervisor was asking them to be quiet, because they were shouting and screaming at each other.' (Patient 4)*

Yet these observations of staff behaviours were quite nuanced. Patients recognised different types of unproductive working relationships, describing over-familiarity (*'almost like a bunch*

For peer review only - http://bmjopen.bmj.com/site/about/guidelines.xhtml

of friends working together' – Patient 3), as well as open antagonism. They also made allowances for the general workload on the ward, even excusing displays of inappropriate behaviour:

> *'There's a lot of pressure put on the staff, you know it's understandable. You can see that they're actually very tired people, they needed a good rest, and that's why the whole thing gets on top of them, they're overworked.' (Patient 4)*

For peer review only

3. Hygiene

In a similar vein, patients and carers expressed quite subtle views of why they held hygiene standards to be important. First, good hygiene was *de facto* evidence of a ward that was providing safe care, with little risk of iatrogenic infection. Patients and carers were conscious of the possibility of hospital-acquired infection, understanding it as a major risk associated with inpatient care. Minimising that risk made it possible to focus on the acute medical issues at hand. Second, good hygiene served as a deeper marker of staff pride, diligence and attention to detail, all of which were reassuring:

> *'The cleanliness aspect, I think, is... more important than possibly people realise... It sets out a marker if you like... if the mindset of the ward is, you know, "We are proud of the place that we work in." So it is a fairly good marker of how that ward will actually be.' (Patient 3)*

How did patients and carers perceive the quality metrics on display?

1. Benefits – using infection data for hand hygiene, and understanding staff performance

For peer review only - http://bmjopen.bmj.com/site/about/guidelines.xhtml

Patients and carers described some benefits of the quality metrics on display, particularly when it came to infection data. They acknowledged prompts to focus on their own hand hygiene, whilst hoping that staff would do the same. In some cases, a vague familiarity with infection control terminology was helpful:

> *'Because that's in the press [MRSA rates], I suppose people do want to know that, don't they? All of this you read in the papers of people being in hospital - they went in with one thing and they came out with that... You don't want to get worse. They're meant to be making you better.' (Patient 5)*

Real-time information on staffing levels was also potentially helpful, in that it could help set realistic expectations of the care patients might receive:

> *'When I'm getting poor service on a particular day, at least I can see that there might be a good reason for it... I would be more understanding, if I had to wait twice as long for help, if I knew that there was only half the number of staff there should be.' (Patient 8)*

> *'When I saw the amount of staff that you're supposed to have on the ward, there were not half the staff. So the other staff that turned up were constantly busy, running back and forth, and you can see how much stress they were [under]. But they were doing a good job... You can see the nurse who has turned up is doing a really good job.' (Carer 1)*

2. Significant drawbacks – problems in information delivery, prioritisation of personal experience, and unintended consequences

For peer review only - http://bmjopen.bmj.com/site/about/guidelines.xhtml

However, patients and carers were largely disparaging about the quality metrics on display. There were numerous problems with information delivery, such as inadequate font size or colour contrast. Yet even with these issues addressed, the information provided was fundamentally inadequate to make a judgement about quality. Patients struggled to see the relevance of a single figure when no trend or benchmark was provided:

> *'Obviously as a member of the public I want the minimum [information], but I have nothing to compare it with. So if you [say], "We've not had one [infection] for three years", I can't compare that with anything. So it doesn't mean anything to me...' (Carer 5)*

> *'That [single figure] doesn't mean anything. That doesn't inform. It could be an increase... but it could be [a] decrease.' (Patient 2)*

More broadly, participants felt little need for measures that related to their wards' previous performance. Their ongoing experiences of care would override any other evaluation. From each individual's perspective, their personal care was the priority, whether or not it reflected a typical standard of care on that ward. In that light, other performance metrics became irrelevant:

> *'I use my own judgement. If I'm satisfied: that's it.' (Patient 9)*

> *'If we want information, we ask for it and we get it. As long as [my relative] is alright and getting looked after, I'm not really bothered about nothing else. If she's getting*

For peer review only

well looked after, the nurses are lovely, their care is great... that's all we are concerned about.' (Carer 3)

As a result, the production of ward quality metrics had some unintended consequences, even going so far as to reduce patients' trust in the whole enterprise. The absence of baseline data in quality displays in particular raised suspicions that poor performance was being concealed.

'Let us face it... you have got your 100% figure there. Would you put up a 20% figure? ... What would you be doing? You would be ruining the confidence of the patients...' (Patient 3)

Patients and carers felt that staff had to have ownership of the quality agenda in hospital: quality metrics were for staff – not patients – to digest. Many interviewees drew comparisons with other settings in which they were consumers: as restaurant diners, or as car purchasers, where their ability to exercise a choice was crucial. Here, however, they had no power to choose, and the display of performance reports only served to emphasise their lack of control:

'It would be great if I'm admitted and I'm given a choice of five wards, and I would say, "Well, how do I know which one's which, which one's best?" My next question would be, "Can you give me the audits of those wards to show which has the highest rating?" and I would go to that... If there's no choice, then it's all academic.' (Patient 8)

For peer review only

'Well, other than clean the wards, there's not a lot we can do is there? What else can you do?' (Carer 4)

The 'Friends and Family Test' question was found to be particularly challenging, given that these patients had no choice in arriving on the ward in the first place, nor could subsequent patients exercise a preference to get there. Indeed, the service pressures on hospital admissions were so well publicised that the idea of choosing a ward seemed faintly ridiculous:

'Would I recommend a ward? How can you recommend a ward?... I mean, that's a daft question, because... they put you in the place you need to be, don't they?' (Patient 5)

DISCUSSION

To our knowledge, this is the first study to compare publicly-displayed performance metrics with patient and carer perceptions of high quality care on UK medical wards. We identified discrepancies between patient- and carer-identified priorities and the quality metrics relating to their care on general medical wards. Patients and carers expressed three core components of high quality general medical care: communication, staff attitudes, and hygiene. These were only partially aligned with the performance measures on display. Specifically, we found process and outcome measures relating to hand hygiene and iatrogenic infection, but none specifically relating to attitudes or communication. Patients and carers acknowledged limited benefits to the display of performance data, but had significant reservations about how it was contextualised. They relied on their own experience of care to judge its quality, above any objective measure of performance. More philosophically, they questioned the purpose of publicly displayed performance data, given

For peer review only

their lack of choice in this setting. In some cases, these reservations actually eroded trust in ward teams' performance.

This study builds on a body of research exploring patient priorities and patient involvement in the acute hospital setting. Boyd surveyed recently-discharged patients, similarly finding that communication, patient-professional interactions, hygiene and the technical delivery of care were their main priorities.[13] Our study suggests that Boyd's findings (which excluded current inpatients) were not unduly affected by recall bias. Nonetheless, hospitalised patients remain relatively indifferent to service-level performance and change.[14] We suggest an explanation for this: current inpatients are unable to exercise informed choices about their ward, nor are they able to directly use information to improve performance. They are therefore excluded from the two key pathways by which performance measurement may lead to quality improvement.[15]

Our findings question the mandatory collection, and display, of performance data that do not align with patient priorities. These data collection exercises have considerable opportunity costs. We note the recent call for the abolition of the mandatory 'Friends and family test', one of the performance indicators we found on display, which has been criticised on similar lines.[16] These data sets are expensive to maintain, *"at best tolerated, often ignored, and sometimes ridiculed"*.[16] The resulting tokenistic display of performance data erodes patients' trust in the system that organises and governs their care. It can also be corrosive for staff morale, both at the frontline and at board level.[17,18] This tokenism is perpetuated by a dearth of resources for implementing meaningful improvement.[1,19] A credible, co-produced, quality framework for acute medical inpatients is urgently required, with outcomes that are sensitive to the work[20] and structures[21] of inpatient care. Co-

For peer review only - http://bmjopen.bmj.com/site/about/guidelines.xhtml

produced quality standards should capitalise on the active contributions of patients and carers, rather than depicting them as 'informed choosers' of healthcare provision.

Study limitations include a relatively small sample from a single site. We collected demographic data for patients but not their carers. Nonetheless, the group of interviewees included demographics often excluded from safety and quality research: older, frail patients, and those who do not speak English as a first language.[22] The study reached data saturation, with no new themes emerging as the final interviews took place. Ward displays at this site were also typical for regional practice. 'Static' performance measures, as seen here, are widespread, and even the performance data presented at healthcare board level rarely depicts the role of chance in the formation of data patterns.[23,24] Finally, other repositories for quality metrics, beyond those ward displays analysed here, may better approximate patient priorities. However, they typically use composites of the data we found,[25] or are aggregated to the hospital level, with no ward-level interpretation.[26,27]

In conclusion, we found a gap between general medical inpatients' care priorities and the aspects of care that are publicly reported. Where performance measurement could have been useful to patients and carers, suboptimal displays only served to emphasise their passive receipt of services. Unless patients and carers are invited to define the quality metrics they hold relevant, ward services may struggle to engage them in improvement efforts. Ultimately, tokenistic quality measurement may have unintended consequences, eroding patients' trust in their healthcare teams.

REFERENCES

1. Lawton R, O'Hara JK, Sheard L, et al. Can patient involvement improve patient safety? A cluster randomised control trial of the Patient Reporting and Action for a Safe Environment (PRASE) intervention. BMJ Qual Saf 2017;**26**(8):622-31.

For peer review only - http://bmjopen.bmj.com/site/about/guidelines.xhtml

2. Shaller D, Kanouse DE, Schlesinger M. Context-based strategies for engaging consumers with public reports about health care providers. Med Care Res Rev 2014;**71**(5 Suppl):17S-37S.
3. Department of Health. Hospital 'friends and family test' announced. Available at https://www.gov.uk/government/news/hospital-friends-and-family-test-announced. Accessed 11/12/2017.
4. Morris D, Karlson A. Dynamic Accessibility Requirements for Hospital Patients. SIGCHI Conference on Human Factors in Computing Systems. Vancouver, Canada: ACM Press, 2011.
5. Pannick S, Wachter RM, Vincent C, et al. Rethinking medical ward quality. BMJ 2016;**355**:i5417.
6. Rose G. On the relation between 'visual research methods' and contemporary visual culture. The Sociological Review 2014;**62**(1):24-46.
7. Schwartz D. Visual ethnography: Using photography in qualitative research. Qualitative Sociology 1989;**12**(2):119-54.
8. Pope C, van Royen P, Baker R. Qualitative methods in research on healthcare quality. Quality and Safety in Health Care 2002;**11**(2):148-52.
9. Tobiano G, Bucknall T, Marshall A, et al. Patients' perceptions of participation in nursing care on medical wards. Scand J Caring Sci 2016;**30**(2):260-70.
10. Sivell S, Prout H, Baillie J, et al. Conducting qualitative research interviews with palliative and end of life care patients in the home setting; reflections and recommendations from an academic palliative care research centre. BMJ Supportive & Palliative Care. 2015;**5**(1):117-17.
11. Sivell S, Prout H, Hopewell-Kelly N, et al. Considerations and recommendations for conducting qualitative research interviews with palliative and end-of-life care patients in the home setting: a consensus paper. BMJ Supportive & Palliative Care 2015;10.1136/bmjspcare-2015-000892.
12. Braun V, Clarke V. Using thematic analysis in psychology. Qualitative Research in Psychology 2006;**3**(2):77-101.
13. Boyd J. The 2006 inpatients importantance study. Oxford: Picker Institute, 2007. http://www.nhssurveys.org/Filestore/documents/Findings_and_development_of_the_2006_Inpatients_Importance_study_final.pdf.
14. Wray CM, Farnan JM, Arora VM, et al. A qualitative analysis of patients' experience with hospitalist service handovers. J Hosp Med 2016;10.1002/jhm.2608.
15. Berwick DM, James B, Coye MJ. Connections between quality measurement and improvement. Med Care 2003;**41**(1 Suppl):I30-8.
16. Robert G, Cornwell J, Black N. Friends and family test should no longer be mandatory. BMJ 2018;**360**:k367.
17. NHS England. Review of the Friends and Family Test. 21st July 2014. Available at https://www.england.nhs.uk/wp-content/uploads/2014/07/fft-rev1.pdf. Accessed 16/10/2017.
18. Raleigh V, Thompson J, Jabbal J, et al. Patients' experience of using hospital services. London: The King's Fund, 2015. http://www.pickereurope.org/wp-content/uploads/2015/12/Patients-experience-Kings-Fund-Dec-2015.pdf.
19. Sheard L, Marsh C, O'Hara J, et al. The Patient Feedback Response Framework - Understanding why UK hospital staff find it difficult to make improvements based on patient feedback: A qualitative study. Soc Sci Med 2017;**178**:19-27.
20. Pannick S, Davis R, Ashrafian H, et al. Effects of Interdisciplinary Team Care Interventions on General Medical Wards: A Systematic Review. JAMA Intern Med 2015;**175**(8):1288-98.

For peer review only - http://bmjopen.bmj.com/site/about/guidelines.xhtml

21. Dumitrascu AG, Burton MC, Dawson NL, et al. Patient portal use and hospital outcomes. Journal of the American Medical Informatics Association 2018;**25**(4):447-53.
22. O'Hara JK, Lawton RJ. At a crossroads? Key challenges and future opportunities for patient involvement in patient safety. BMJ Qual Saf 2016;10.1136/bmjqs-2016-005476.
23. Anhoj J, Hellesoe AB. The problem with red, amber, green: the need to avoid distraction by random variation in organisational performance measures. BMJ Qual Saf 2016;10.1136/bmjqs-2015-004951.
24. Schmidtke KA, Poots AJ, Carpio J, et al. Considering chance in quality and safety performance measures: an analysis of performance reports by boards in English NHS trusts. BMJ Qual Saf 2017;**26**(1):61-69.
25. NHS Quality Observatory. NHS Safety Thermometer. Available at https://www.safetythermometer.nhs.uk/index.php?option=com_content&view=article&id=1&Itemid=101. Accessed 24th June 2016.
26. Care Quality Commission. NHS Surveys: focused on patients' experience. Available at http://www.nhssurveys.org/. Accessed 23rd June 2016.
27. Centers for Medicare and Medicaid Services. HCAHPS Fact Sheet. June 2015. Baltimore, MD. Available at http://www.hcahpsonline.org/facts.aspx. Accessed 23rd June 2016.
28. NHS England. Friends and Family Test: Guidance. 2014. Publications Gateway Ref No. 01787.

For peer review only

Table 1: Performance indicators available in public ward areas

Performance indicator	*Display choice*	*Display format*
Hand hygiene	Percentage in last audit	Most recent result only; numerator and denominator definitions not provided.
Hospital-acquired infections	Date of last recorded event	Most recent result only
Pressure ulcers	Date of last recorded event	Most recent result only
Falls	Date of last recorded event	Most recent result only
Nurse staffing	Numbers of staff required for the shift *vs* those actually on duty, for staff nurses and health care assistants	Most recent result only; explanation of staff responsibilities
Patient feedback	'Friends and Family Test' star rating; percentage of patients who would recommend the ward*	Most recent result only; examples of patients' comments; no explanation of star rating system

*The 'Friends and Family Test' asks *"How likely are you to recommend our service to friends and family if they needed similar care or treatment?"*[28]

For peer review only

TOPIC GUIDE: Current patient / carer for current patient

1. Let's find out about you.

Age
Employment
Education – school / university / postgraduate
Social support structures & marital status
Ethnicity

2. Why are you in hospital now?

Current diagnosis
Other conditions
Approximate length of stay to date
< 1 day
1 – 5 days
5 – 10 days
> 10 days

3. How many times have you been admitted to hospital in the last 6 months?

1-5
5-10
>10

4. Do you always come to this hospital or have you been admitted to other local hospitals?

5. How do you know if you're on a good ward? What is a 'good ward' to you?

Environment
- Clean
- Quiet
- Toilet and shower are available when required
- Meal timeliness, warmth
- Help available when requested
- Staff are responsiveness to my needs / my family's needs

Welcome
- My arrival is expected
- Staff introduce themselves
- Staff make me feel I will be well looked after; show a caring attitude; and don't rush me

Communication and use of personal information
- Accurate knowledge of previous medical history / current diagnosis / current investigations / discharge plan / medication reconciliation
- Quality of communication / teamwork

Discharge preparation

For peer review only

Friends' / families' recommendations

Ward information boards / quality and safety boards

Ward information leaflets / other printed materials.

Ward information displays / electronic screens

6. **If you had to decide whether a ward was good or not, what information would you need to make that decision?**

7. **Have you noticed any of the information the ward displays about itself? What do you think of the information you've seen?**

Friends and family test results
Safety cross
Shift-by-shift staffing
Falls
Pressure ulcers
Safety thermometer / harm-free care
Venous thromboembolism prophylaxis
Hand hygiene compliance
Hospital-acquired infections
Infection rates
Incident reporting

8. **What would you like to know about how your ward is performing?**

Hand hygiene compliance
Staffing levels
Friends and family results
Hospital-acquired infections
Pressure Ulcers
Falls
Venous thromboembolism prophylaxis
Complaints
Compliments
Length of stay
Mortality
Readmission rate
Safety climate

9. **How should your ward make that information available to you and your family??**

Ward displays
Leaflets
Smartphone / other device
Webpage

For peer review only

10. Preference for information seeking

Information-seeking sub-scale

	Disagree strongly	Disagree slightly	Neutral	Agree slightly	Agree strongly
As you become sicker you should be told more and more about your illness					
You should understand completely what is happening inside your body as a result of your illness					
Even if the news is bad, you should be well informed					
Your doctor should explain the purpose of your laboratory tests					
It is important for you to know all the side effects of your medication					
Information about your illness is as important to you as treatment					
When there is more than one method to treat a problem, you should be told about each one					

11. Have you previously had to complain about care or healthcare staff, nurses or doctors? What made you complain? How? PALS / informally / in writing?

For peer review only

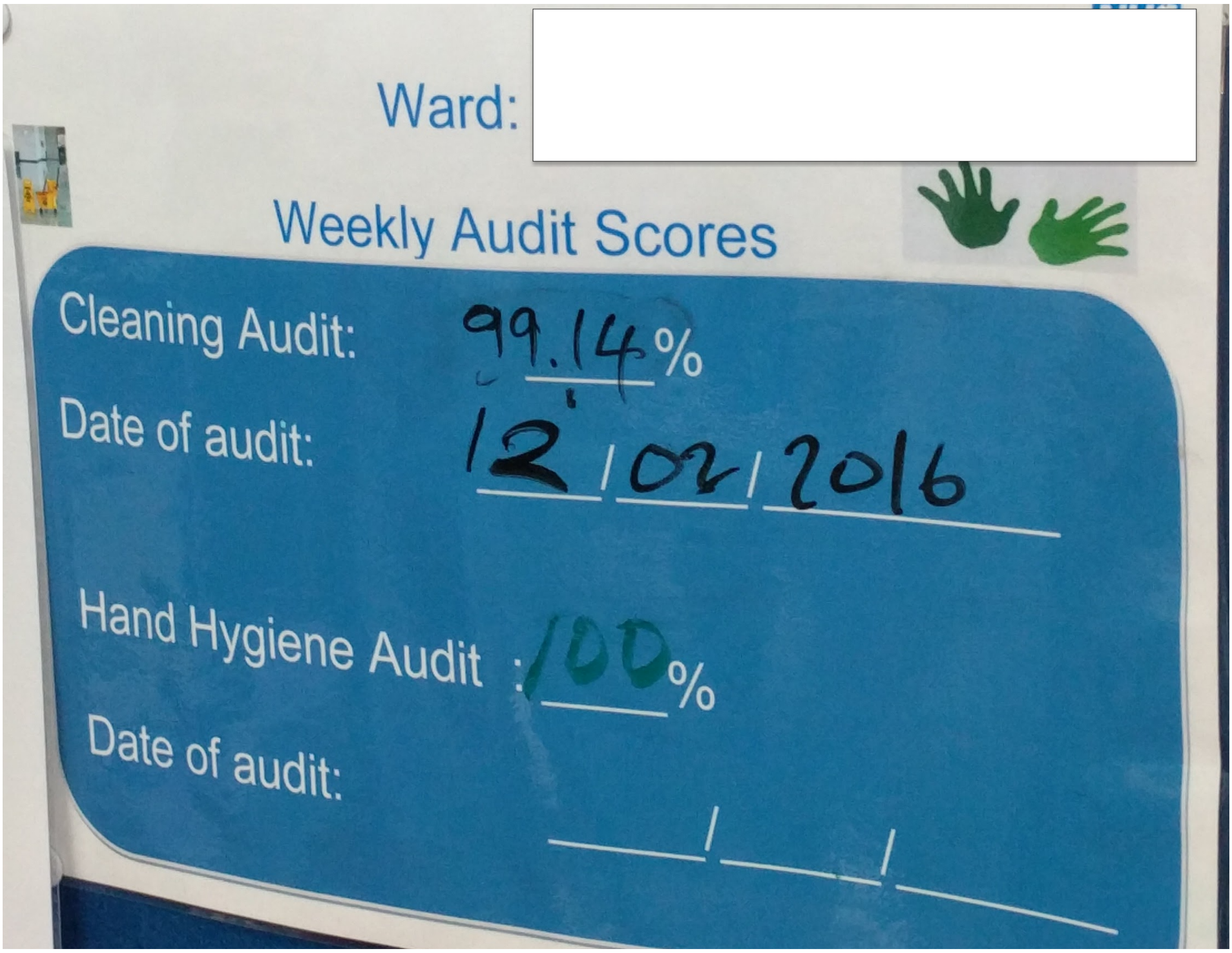
Ward:
Weekly Audit Scores
Cleaning Audit: 99.14%
Date of audit: 12/02/2016
Hand Hygiene Audit : 100%
Date of audit: / /

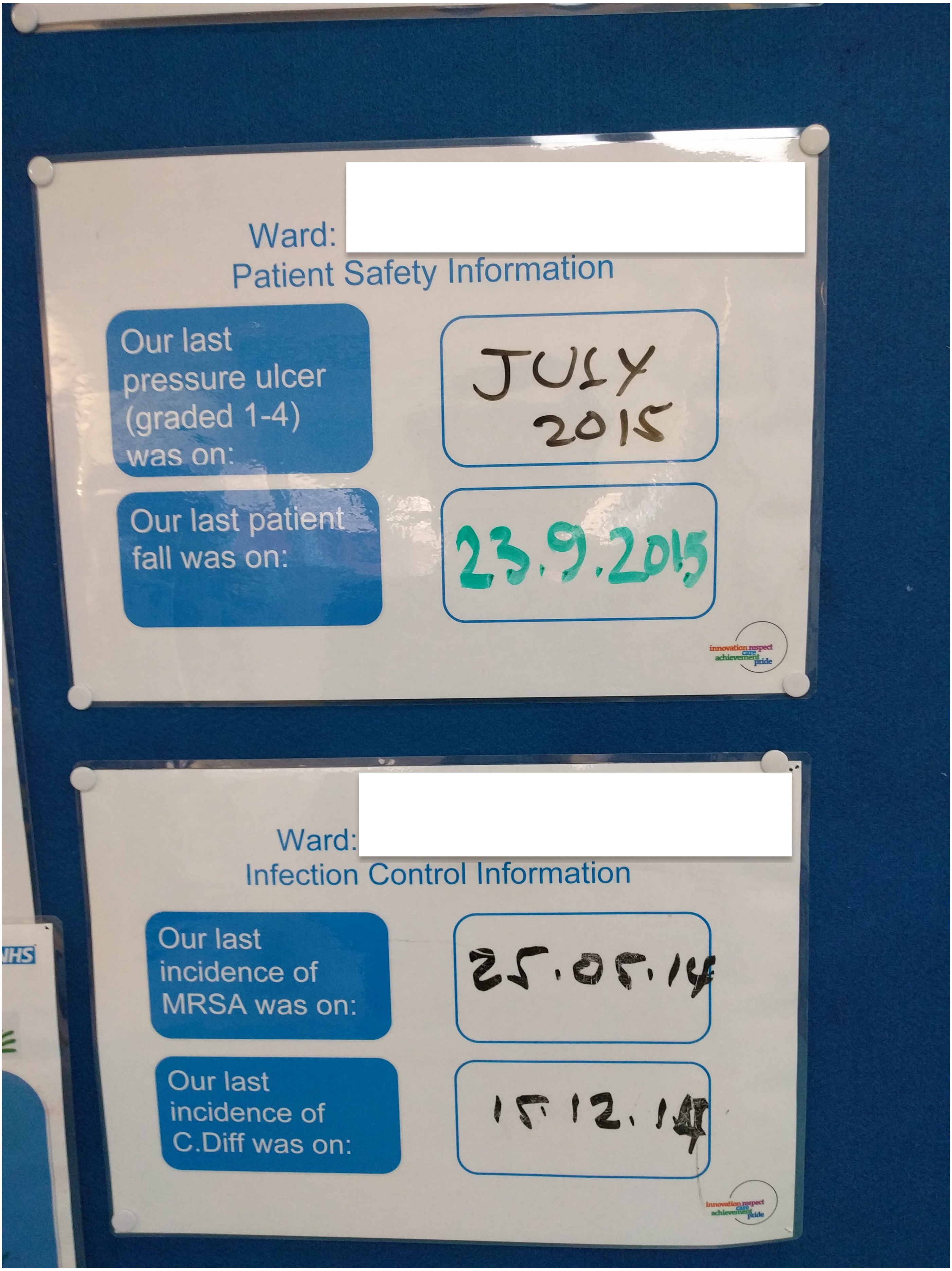
Ward:
Patient Safety Information
Our last pressure ulcer (graded 1-4) was on:
JULY 2015
Our last patient fall was on:
23.9.2015
Ward:
Infection Control Information
Our last incidence of MRSA was on:
25.05.14
Our last incidence of C.Diff was on:
innovation respect care achievement pride

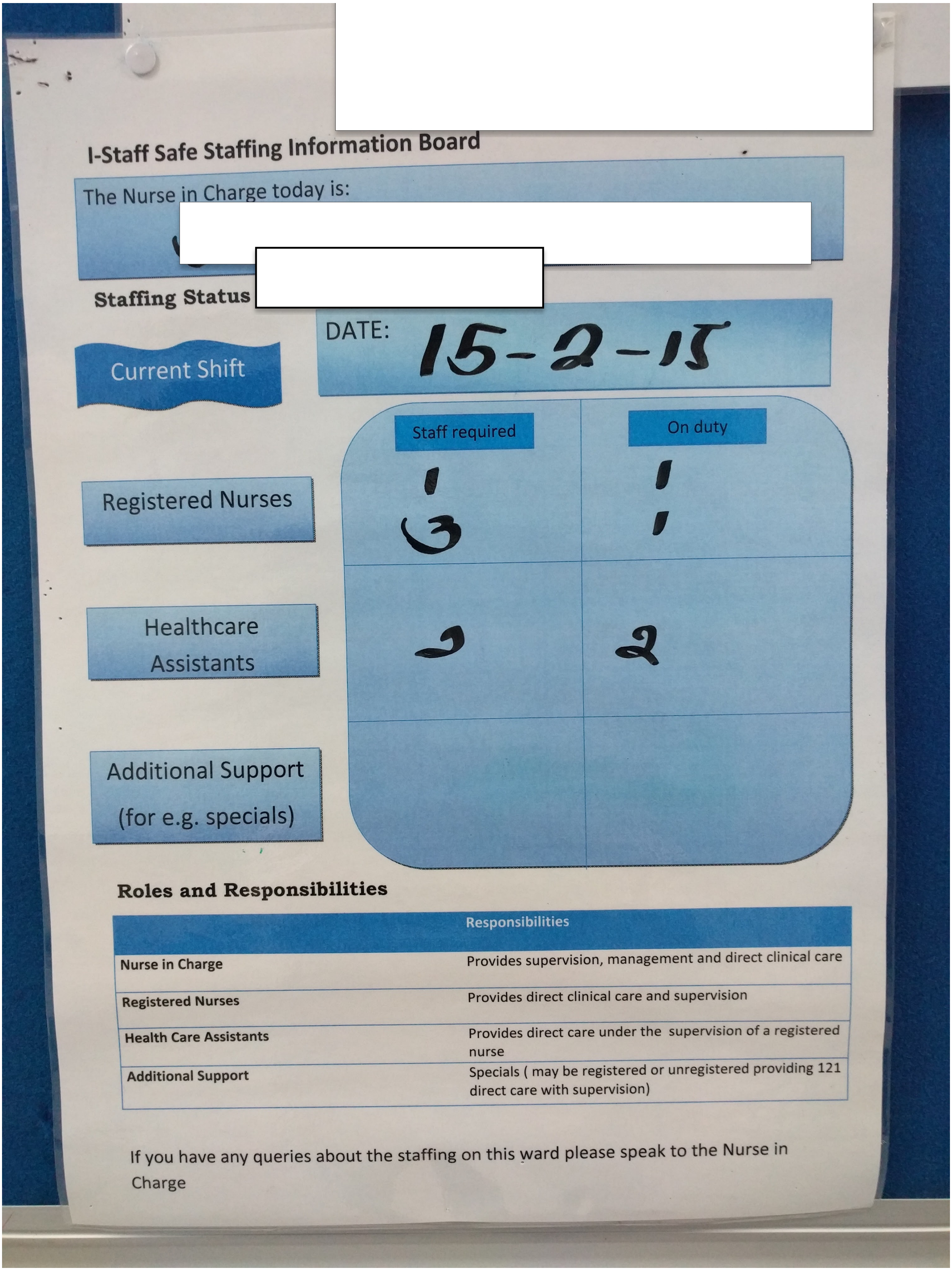
I-Staff Safe Staffing Information Board
The Nurse in Charge today is:
Staffing Status
Current Shift
DATE: 15-2-15
Staff required
On duty
Registered Nurses
1
3
1
1
Healthcare Assistants
2
2
Additional Support (for e.g. specials)
Roles and Responsibilities
Responsibilities
Nurse in Charge
Provides supervision, management and direct clinical care
Registered Nurses
Provides direct clinical care and supervision
Health Care Assistants
Provides direct care under the supervision of a registered nurse
Additional Support
Specials (may be registered or unregistered providing 121 direct care with supervision)
If you have any queries about the staffing on this ward please speak to the Nurse in Charge

COREQ (COnsolidated criteria for REporting Qualitative research) Checklist

A checklist of items that should be included in reports of qualitative research. You must report the page number in your manuscript where you consider each of the items listed in this checklist. If you have not included this information, either revise your manuscript accordingly before submitting or note N/A.

Topic	Item No.	Guide Questions/Description	Reported on Page No.
**Domain 1: Research team and reflexivity**			
Personal characteristics			
Interviewer/facilitator	1	Which author/s conducted the interview or focus group?	7
Credentials	2	What were the researcher's credentials? E.g. PhD, MD	7
Occupation	3	What was their occupation at the time of the study?	7
Gender	4	Was the researcher male or female?	7
Experience and training	5	What experience or training did the researcher have?	7
Relationship with participants			
Relationship established	6	Was a relationship established prior to study commencement?	7
Participant knowledge of the interviewer	7	What did the participants know about the researcher? e.g. personal goals, reasons for doing the research	7
Interviewer characteristics	8	What characteristics were reported about the inter viewer/facilitator? e.g. Bias, assumptions, reasons and interests in the research topic	7
**Domain 2: Study design**			
Theoretical framework			
Methodological orientation and Theory	9	What methodological orientation was stated to underpin the study? e.g. grounded theory, discourse analysis, ethnography, phenomenology, content analysis	7
Participant selection			
Sampling	10	How were participants selected? e.g. purposive, convenience, consecutive, snowball	7
Method of approach	11	How were participants approached? e.g. face-to-face, telephone, mail, email	7
Sample size	12	How many participants were in the study?	7
Non-participation	13	How many people refused to participate or dropped out? Reasons?	7
Setting			
Setting of data collection	14	Where was the data collected? e.g. home, clinic, workplace	7
Presence of non-participants	15	Was anyone else present besides the participants and researchers?	7
Description of sample	16	What are the important characteristics of the sample? e.g. demographic data, date	8
Data collection			
Interview guide	17	Were questions, prompts, guides provided by the authors? Was it pilot tested?	6
Repeat interviews	18	Were repeat inter views carried out? If yes, how many?	7
Audio/visual recording	19	Did the research use audio or visual recording to collect the data?	7
Field notes	20	Were field notes made during and/or after the inter view or focus group?	7
Duration	21	What was the duration of the inter views or focus group?	8
Data saturation	22	Was data saturation discussed?	7
Transcripts returned	23	Were transcripts returned to participants for comment and/or	7

For peer review only

Topic	Item No.	Guide Questions/Description	Reported on Page No.
		correction?	
**Domain 3: analysis and findings**			
Data analysis			
Number of data coders	24	How many data coders coded the data?	7
Description of the coding tree	25	Did authors provide a description of the coding tree?	7
Derivation of themes	26	Were themes identified in advance or derived from the data?	7
Software	27	What software, if applicable, was used to manage the data?	7
Participant checking	28	Did participants provide feedback on the findings?	7
Reporting			
Quotations presented	29	Were participant quotations presented to illustrate the themes/findings? Was each quotation identified? e.g. participant number	9
Data and findings consistent	30	Was there consistency between the data presented and the findings?	9
Clarity of major themes	31	Were major themes clearly presented in the findings?	9
Clarity of minor themes	32	Is there a description of diverse cases or discussion of minor themes?	9

Developed from: Tong A, Sainsbury P, Craig J. Consolidated criteria for reporting qualitative research (COREQ): a 32-item checklist for interviews and focus groups. *International Journal for Quality in Health Care*. 2007. Volume 19, Number 6: pp. 349 – 357

Once you have completed this checklist, please save a copy and upload it as part of your submission. DO NOT include this checklist as part of the main manuscript document. It must be uploaded as a separate file.

For peer review only
